# Loss of BRCA1 or BRCA2 markedly increases the rate of base substitution mutagenesis and has distinct effects on genomic deletions

**DOI:** 10.1038/onc.2016.243

**Published:** 2016-07-25

**Authors:** J Zámborszky, B Szikriszt, J Z Gervai, O Pipek, Á Póti, M Krzystanek, D Ribli, J M Szalai-Gindl, I Csabai, Z Szallasi, C Swanton, A L Richardson, D Szüts

**Affiliations:** 1 Institute of Enzymology, Research Centre for Natural Sciences, Hungarian Academy of Sciences, Budapest, Hungary; 2 Department of Physics of Complex Systems, Eötvös Loránd University, Budapest, Hungary; 3 Center for Biological Sequence Analysis, Department of Systems Biology, Technical University of Denmark, Lyngby, Denmark; 4 Computational Health Informatics Program (CHIP), Boston Children's Hospital, Boston, MA, USA; 5 Harvard Medical School, Boston, MA, USA; 6 MTA-SE-NAP, Brain Metastasis Research Group, 2nd Department of Pathology, Semmelweis University, Budapest, Hungary; 7 CRUK Lung Cancer Centre of Excellence, UCL Cancer Institute, London, UK; 8 Francis Crick Institute, London, UK; 9 Sibley Pathology Department, Department of Pathology, Johns Hopkins Medicine, Baltimore, MD, USA

## Abstract

Loss-of-function mutations in the *BRCA1* and *BRCA2* genes increase the risk of cancer. Owing to their function in homologous recombination repair, much research has focused on the unstable genomic phenotype of *BRCA1/2* mutant cells manifest mainly as large-scale rearrangements. We used whole-genome sequencing of multiple isogenic chicken DT40 cell clones to precisely determine the consequences of *BRCA1/2* loss on all types of genomic mutagenesis. Spontaneous base substitution mutation rates increased sevenfold upon the disruption of either *BRCA1* or *BRCA2*, and the arising mutation spectra showed strong and specific correlation with a mutation signature associated with *BRCA1/2* mutant tumours. To model endogenous alkylating damage, we determined the mutation spectrum caused by methyl methanesulfonate (MMS), and showed that MMS also induces more base substitution mutations in *BRCA1/*2-deficient cells. Spontaneously arising and MMS-induced insertion/deletion mutations and large rearrangements were also more common in *BRCA1/2* mutant cells compared with the wild-type control. A difference in the short deletion phenotypes of *BRCA1* and *BRCA2* suggested distinct roles for the two proteins in the processing of DNA lesions, as *BRCA2* mutants contained more short deletions, with a wider size distribution, which frequently showed microhomology near the breakpoints resembling repair by non-homologous end joining. An increased and prolonged gamma-H2AX signal in MMS-treated *BRCA1/2* cells suggested an aberrant processing of stalled replication forks as the cause of increased mutagenesis. The high rate of base substitution mutagenesis demonstrated by our experiments is likely to significantly contribute to the oncogenic effect of the inactivation of *BRCA1* or *BRCA2*.

## Introduction

An unstable genome is a hallmark of cancer.^1^ Genomic instability in cancer may be caused by the failure of a number of DNA repair and DNA damage tolerance pathways including homologous recombination (HR). The BRCA1 and BRCA2 proteins both have critical roles in HR,^2^ and mutation carriers in the encoding genes are burdened with an elevated risk of breast and ovarian cancer.^3, 4^ BRCA1 promotes DNA end resection at double-strand breaks (DSBs), shifting the balance toward repair by HR rather than non-homologous end joining (NHEJ). The large mediator protein BRCA2 assists the loading of the essential HR factor RAD51 onto RPA-coated single-stranded DNA.^5^ BRCA1 also appears to have an indirect function in RAD51 loading, promoting the recruitment of BRCA2 through their mutual interactions with PALB2.^6^ BRCA1- or BRCA2-deficient tumours display characteristic genomic features bearing evidence of large-scale genome instability: a high level of loss-of-heterozygosity, telomeric allelic imbalance and large-scale state transitions.^7, 8, 9^ These properties are being developed as a predictive clinical diagnostic test.^10^ It is an important question whether *BRCA1/2* mutant cells also acquire excess point mutations, which could contribute to the tumorigenic effect of *BRCA1/2* loss. A *BRCA1/2* defect-specific point mutation spectrum that shows a broad range of mutation types has been inferred from unsupervised pattern-finding in cancer genomes.^11^ To obtain direct causative evidence for the mutagenic effect of *BRCA1/2* loss-of-function, including accurate measurements of the mutation load, experiments in isogenic *BRCA1/2* mutant and non-mutant cell lines or transgenic animals are necessary.

In this study we investigate genomic mutations arising in two different contexts: under normal cell culture or under conditions designed to accelerate one class of endogenous mutagenic processes with methyl methanesulfonate (MMS) treatments. During normal cell growth genomic DNA is subjected to a range of damaging influences; most importantly endogenous reactive oxygen species, endogenous alkylating agents and the spontaneous deamination or hydrolysis of DNA bases.^12, 13^ MMS is a methylating agent that models a major class of endogenous DNA damage primarily via generating N7-methylguanine and N3-methyladenine in similar proportions to the most common endogenous methylating agent S-adenosylmethionine.^14, 15^ MMS does not induce DSBs directly.^16^ Instead, the collapse of replication forks at single-strand breaks generated by base excision repair at MMS lesions may lead to DSBs.^17^ A similar mechanism may give rise to DSBs in untreated cells, explaining the essential function of RAD51 following DNA replication.^18^

Incorrect repair of DSBs is expected to result in short deletions and structural rearrangements, as observed in *BRCA1/2*-deficient cells, but it is not known whether BRCA1 or BRCA2 influences the generation of base substitution mutations by various DNA adducts. Through the genome sequence analysis of mock-treated and MMS-treated chicken DT40 cell clones we show that *BRCA1* or *BRCA2* deficiency strongly and indistinguishably increases the level of spontaneously arising base changes with a mutation spectrum very similar to that observed in *BRCA1/2-*deficient cancer, and also increases mutagenesis induced by high doses of mutagens. The number of insertion/deletion (indel) mutations and rearrangements is also higher in HR mutants, with important differences between *BRCA1* and *BRCA2*, suggesting that point mutations and indels are generated by different mechanisms in HR-deficient cells.

## Results

### Loss of BRCA1 or BRCA2 increases the spontaneous mutation rate

We set out to determine the quantity and map the profile of spontaneous mutations arising in wild-type (WT) and *BRCA1* or *BRCA2* mutant cells. We used a chicken DT40 cell line carrying a homozygous *BRCA1* mutation that deletes exons 6-8, removing the RING domain and eliminating the downstream transcript;^19^ and a *BRCA2* mutant cell line in which one allele is missing the entire coding sequence, whereas the other allele is missing exons 1–2.^20^ A reduced recruitment of RAD51 to sites of damage and hypersensitivity to the PARP inhibitor olaparib confirms the *BRCA1/2* loss-of-function.^20, 21^ Cultures were grown from single cell clones for ~100 cell divisions, when single cell clones were isolated again from the bulk culture. Whole-genome sequences were obtained from the starting clones as well as the clones isolated after mock treatment (for the experimental layout see Supplementary Figure S1).

To extract mutation information, we employed the IsoMut method that performs a simultanous comparison of many samples for efficient noise filtering, whereas achieving a detection rate of ~90% at 20–30 × coverage.^22^ We tuned IsoMut to detect maximum five false-positive single-nucleotide variations (SNVs) per starting clone (Table 1).

**Figure 1:**
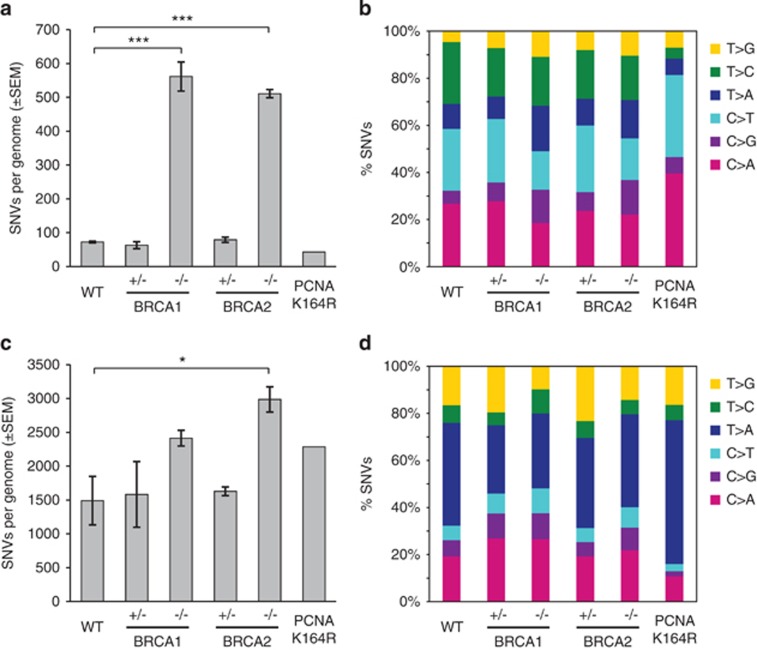
Number and spectrum of SNVs. (**a** and **c**) The mean number of SNVs detected following mock treatment (**a**) or four rounds of weekly 1 h treatments with 20 ppm MMS (**c**) of cell lines of the indicated genotypes. Error bars indicate s.e.m. Significance values are indicated (unpaired *t*-test, *P<0.05, ***P<0.001). (**b** and **d**) Base substitution spectrum of mutations detected following mock treatment (**b**) or MMS treatment (**d**).

**Figure 2:**
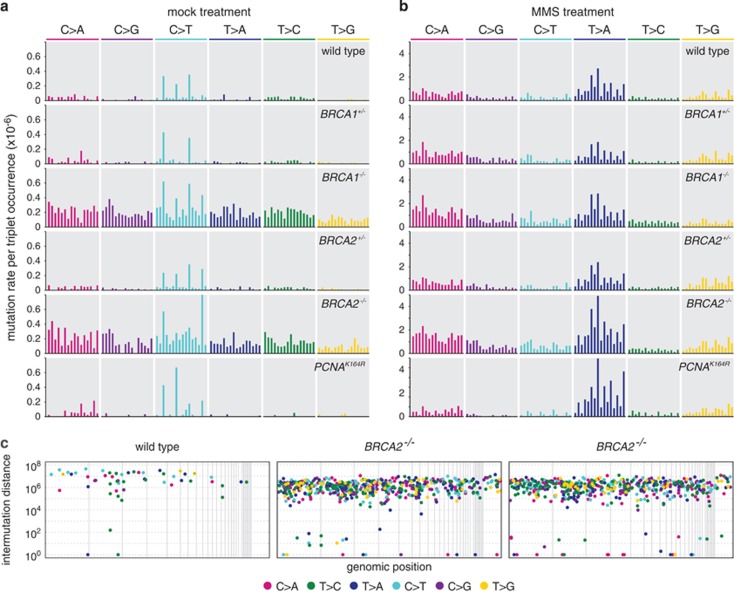
Triplet mutation spectrum and genomic distribution of SNVs. Triplet mutation spectra of the mock treatment (**a**) or MMS treatment (**b**) of the indicated cell lines. Each mutation class, as indicated at the top of the panel, is separated into 16 categories based on the identity of the preceding and following nucleotide. The mutation rate at each triplet was obtained by dividing the number of observed mutations with the number of occurrences of that particular triplet in the chicken genome. The sequence of triplets is shown on expanded Supplementary figures S2-S5; the four C>T peaks in mock-treated samples represent NCG>NTG mutations. (**c**) In mock-treated clones of the indicated genotypes, the distance of each SNV mutation from the previous SNV on the same chromosome is plotted against the genomic position of the mutation. Thin vertical lines indicate chromosome boundaries. Chromosomes are shown in numerical order, chromosome Z is shown last on the right. The colour of each dot illustrates the type of mutation according to the key at the bottom of the panel. One sequenced clone of each cell line is shown.

In the WT mock-treated samples we found 72±5 (s.d.) SNVs, of which C>T changes were most common, followed by C>A (Figures 1a and b). When SNVs are viewed in the context of the neighbouring bases and their frequencies are normalized to the frequency of occurrence of each triplet in the genome, the most commonly mutated triplets were NCG (Figure 2), and NCG>NTG mutations occurred with a 11 × increased likelihood compared with the mean mutation rate. This demonstrates that the cultured cell line used in this study faithfully reproduces the main spontaneous mutagenic process observed in vertebrate genomes, namely C>T changes owing to the deamination of 5-methyl-cytosine at CpG sites.^23^

In contrast to the low number of mutations arising in WT cells, we found a seven- to eightfold higher level of newly generated SNVs in the mock-treated homozygous *BRCA1*^*−/−*^ and *BRCA2*^*−/−*^ samples (Table 1; *P*<0.001, unpaired *t*-test). This elevation of the point mutation rate was due to a massive increase of all six types of base substitutions, with no major shift in their proportions (Figure 1b). The frequency of NCG>NTG mutations did not significantly increase (Figure 2a), whereas there was an increase in all other types of triplet mutations. Our data show that loss of BRCA1 or BRCA2 function strongly and uniformly increases the spontaneous genomic base substitution mutation rate. The mutations appeared unclustered, with fewer than 5% of SNVs within 100 bp of the previous SNV both in WT and *BRCA1/2* mutant samples (Figure 2c), indicating that most mutations arose as independent events.

In the light of reports on *BRCA1* and *BRCA2* haploinsufficiency phenotypes in replication stress and DNA repair^24, 25, 26, 27^ we also assayed the spontaneous mutation rate in *BRCA1*^*+/−*^ and *BRCA2*^*+/−*^ heterozygous cell lines. In both of these, the number of SNVs generated by mock treatment was almost identical to that in the WT (Figure 1a, Table 1). Thus, we observed no haploinsufficiency in the identified function of the *BRCA1/2* genes that protects against somatic base substitution mutations in unstressed normal growth conditions.

### Treatment with an alkylating agent accelerates the mutagenic process

**Figure 3:**
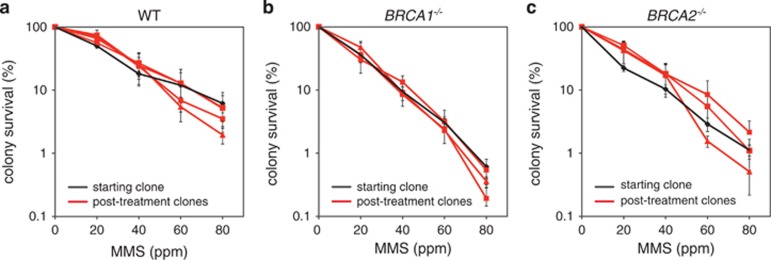
MMS sensitivity of pre-treatment and post-treatment clones. Colony survival assays measuring the sensitivity of WT (**a**), *BRCA1*^*−/−*^ (**b**) and *BRCA2*^*−/−*^ (**c**) cell lines to MMS. In each panel, the pre-treatment starting cell clone (black) and the three sequenced post-treatment clones are shown. Error bars indicate s.e.m.

In contrast to spontaneous mutations attributable to a range of DNA lesions and cellular processes, treatment with defined DNA-damaging agents should produce a specific set of DNA lesions and elicit a specific mutation pattern. To understand the role of BRCA1/2 defects in increased base substitution mutagenesis, we treated the experimental cell lines with the methylating agent MMS, selecting a concentration (20 ppm, 236 μm) which kills ~50% of the cell population (Figure 3a). As BRCA1- and BRCA2-deficient cells are hypersensitive to MMS, lower survival was seen with the homozygous knockout cell lines (36% and 22%, respectively; Figures 3b and c). A bulk cell population was subjected to four weekly rounds of treatment with MMS, with the same overall timing as the mock treatments (Supplementary Figure S1). MMS sensitivity measurements of starting clones and post-treatment clones indicated that the treatment regimen did not result in the selection of cells that developed resistance (Figures 3a and c).

Treatment of WT DT40 cells with MMS accurately revealed, for the first time, the spectrum of mutations induced by this DNA-damaging agent. The total number of SNVs increased over 20-fold (Figures 1a and c, Table 1), and all six base substitution categories were more frequent than after mock treatment, with T>A and C>A mutations the most common (Figure 1d). The abundance of T>A mutations, which were most abundant in triplets containing further pyrimidines (Figure 2b, Supplementary Figures S2–S5) suggests that the major mutagenic effect of MMS is the consequence of adenine methylation.

The number of base substitution mutations in MMS-treated *BRCA1*^*−/−*^ and *BRCA2*^*−/−*^ homozygous mutants (Table 1) was greater than in the WT (Figure 1c, Table 1), though this difference was only significant for BRCA2 (*P*=0.070 and *P*=0.021, respectively). Again, we observed no increased mutagenesis in the *BRCA1*^*+/−*^ and *BRCA2*^*+/−*^ heterozygous cell lines (Figure 1c, Table 1). The difference between WT and *BRCA1/2* knockout mutants after MMS treatment is 2–3 times greater than after mock treatment, therefore even after subtraction of spontaneous mutations, MMS induces more SNVs in *BRCA1/2* mutant cells than in the WT. Interestingly, the spectrum of SNVs in the MMS-treated WT and *BRCA1/2* mutant samples is nearly identical (Figures 1d and 2b). This suggests that the disruption of *BRCA1* or *BRCA2* does not affect the actual mutagenic process, rather influencing how frequently it is employed.

A major mutagenic cellular process in proliferating cells is translesion DNA synthesis (TLS).^28^ TLS is performed by specialized translesion polymerases, several of which can be recruited to DNA via binding to the monoubiquitylated form of the essential replication protein proliferating cell nuclear antigen (PCNA).^29^ TLS can also take place in the absence of PCNA monoubiquitylation via the recruitment of TLS polymerases by REV1,^30^ though with an altered mutagenic profile.^31, 32^

We tested the contribution of TLS to MMS-induced mutagenesis using the *PCNA*^*K164R*^ cell line in which PCNA ubiquitylation is not possible.^33^ In this cell line the number of spontaneous mutations was not significantly greater than in the WT (Figure 1b). However, the sequencing of a cell clone after MMS treatment revealed an important change in the mutation spectrum compared with the WT and the *BRCA* mutants, showing a selective increase in A>T mutations, and a reduction of several other mutation classes (Figures 1d and 2b). This result suggests that the MMS-induced mutations in the WT, and the identical MMS mutation spectrum in the *BRCA1* and *BRCA2* mutants is indeed shaped by the process of TLS.

### The analysis of short insertions and deletions reveals differences between BRCA1- and BRCA2-associated mutagenesis

**Figure 4:**
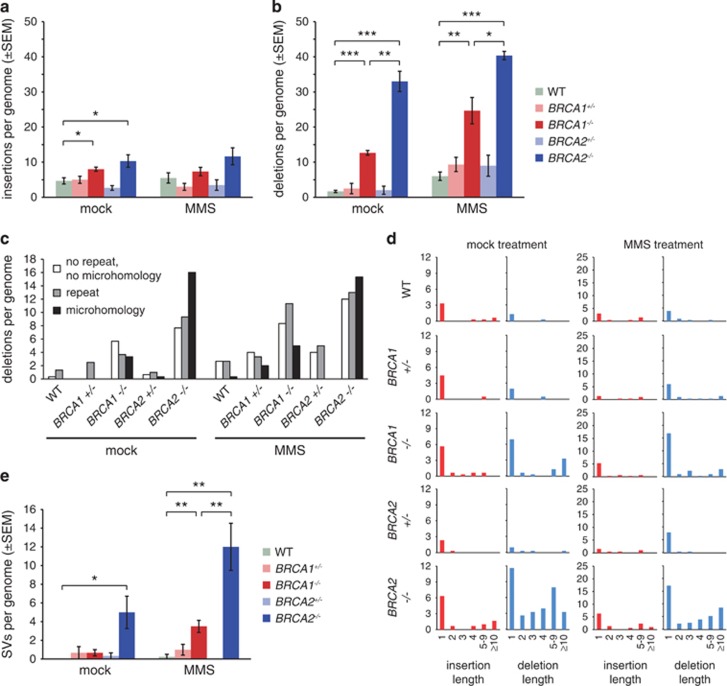
Indels and large rearrangements. (**a** and **b**) The mean number of detected short insertions (**a**) or deletions (**b**) that arose following mock treatment or MMS treatment of cell lines of the indicated genotypes. (**c**) The mean number of short deletions per genome classified according to the sequence of the deletion and its context. The minimum length of classified microhomologies was 1 bp. (**d**) The mean number of insertions and deletions per genome of the indicated genotypes, categorized by length as indicated at the bottom of the panel. Left columns, mock treatment; right columns, MMS treatment. (**e**) The mean number of structural variations (SV) per genome, including larger insertions, deletions, duplications and other structural rearrangements. Error bars indicate s.e.m. Significance values are indicated (unpaired *t*-test, *P<0.05, **P<0.01, ***P<0.001).

BRCA mutations have been associated with the increased appearance of indels in tumour genomes.^34^ We used the IsoMut mutation detection method to identify short indels up to 50 bp.^22^ In WT mock-treated samples we found 4.7±1.5 (s.d.) spontaneously arising insertions and 1.7±0.6 deletions per genome (Table 1, Figures 4a and b). In contrast, genomes of *BRCA1*^*−/−*^ mock-treated samples contained 8.0±1.0 short insertions and 12.7±1.2 deletions, a significant increase in each mutation category (*P*=0.034 and *P*<0.001, respectively) and altogether a fourfold increase in indels compared with the WT. *BRCA2*^*−/−*^ mock-treated samples contained eight times more indels than the WT, with a significant increase in both the number of insertions (10.3±3.1, *P*=0.045) and deletions (40.3±2.1, *P*<0.001). There was no significant difference between the numbers of spontaneous short indels in the WT sample and the *BRCA1*^*+/−*^ and *BRCA2*^*+/−*^ heterozygotes (Figures 4a and b). MMS treatment further increased the number of deletions in all investigated cell lines, while it had no significant effect on insertion mutations (Figures 4a and b).

To better understand the processes generating insertions and deletions, we examined the length distribution and sequence context of indels. In the WT and the *BRCA1/2* heterozygous lines most indels were one-base long. In contrast, we found a distinct length pattern in each BRCA mutant cell line. In *BRCA1*^*−/−*^ cells, 1 bp and over 10 bp long deletions were most common, whereas in *BRCA2*^*−/−*^ there was a significantly different, broader distribution of various indel lengths (Figure 4d, *P*=0.037, Kolmogorov–Smirnov test). MMS treatment typically doubled the number of deletions, but maintained the difference between *BRCA1*^*−/−*^- and *BRCA2*^*−/−*^-specific indel length distributions (*P*=0.010, Kolmogorov–Smirnov test), suggesting that similar processes cause indels at spontaneous or MMS-derived DNA lesions. There were significantly more deletions in *BRCA2*^*−/−*^ samples than in *BRCA1*^*−/−*^ samples (*P*=0.002 and *P*=0.017 in mock- and MMS-treated samples, respectively, Figure 2b). We classified deletions according to their sequence context. In general, there was an increase in all categories in the *BRCA1/2* mutants compared with the WT, but the distribution of the deletions between the three applied categories was also significantly different (*P*=0.024 and *P*=0.026 for mock-treated and MMS-treated samples, respectively, Fisher's exact test). Most notable was the increase (fivefold in mock-treated and threefold in MMS-treated samples) in *BRCA2*^*−/−*^ mutants in deletions displaying evidence of microhomology, that is, partial repetition of 1–5 bases at the end of these deletions (Figure 3c). Such short microhomologies at deletions are likely evidence of DNA double-strand break repair by NHEJ, particularly its microhomology-mediated variety.^35^ Taken together, the greater increase in deletion numbers, the broader range of deletion length and the higher proportion of microhomology-derived deletions suggest that certain DNA lesions in the absence of BRCA2 are frequently repaired by NHEJ, whereas in the absence of BRCA1 these lesions may have alternative modes of repair. A search for larger indels, duplications and translocations revealed a significant increase in both spontaneous and MMS-induced larger scale genomic rearrangements in *BRCA2*^*−/−*^ cells (Figure 3e), again supporting the view that failure of error-free HR in the absence of BRCA2 results in the increased use of error-prone NHEJ or other repair mechanisms.

### The BRCA1/2 spontaneous mutation spectrum is present in tumour genome sequences

**Figure 5:**
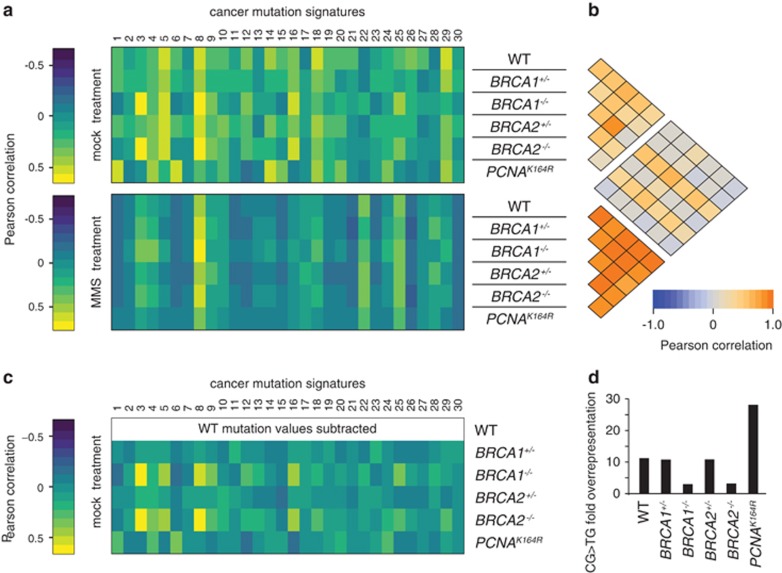
Correlation of detected SNV patterns with mutation spectra identified in cancer. (**a**) Heat map of the Pearson correlation coefficient between triplet base mutation patterns induced by mock treatment (top panel) or MMS treatment (bottom panel) in cell lines of the indicated genotypes, and the 30 confirmed mutational signatures identified in human cancer. The heat map keys are shown on the left. (**b**) Heat map showing Pearson correlation coefficients between each pair of cell line and treatment specific mutational patterns. (**c**) Heat map of the Pearson correlation coefficient between the spontaneous mutation patterns of each indicated cell line after the subtraction of the mean WT mutation values, and the cancer mutation signatures as in (**a**). (**d**) Fold overrepresentation of CG>TG base substitutions in mean mutation data sets of mock-treated samples.

A specific SNV spectrum, termed a mutagenic signature, has been associated with *BRCA1/2* gene defects in tumour samples.^11^ When we compared all 30 currently documented tumour mutation signatures with the mean observed spontaneous mutation pattern of each cell line, it was apparent that the mutation pattern in the *BRCA1*^*−/−*^ and *BRCA2*^*−/−*^ cell line samples showed strongest correlation with the BRCA1/2 tumour associated ‘signature 3' (Figure 5a) and with each other (Figure 5b). Other samples, such as the *PCNA*^*K164R*^ spontaneous mutation pattern, did not correlate with this tumour signature. This correlation was even more apparent when we subtracted from all other data sets the WT triplet mutation frequencies, which may represent common mutational processes operating in each cell line (Figure 5c). The SNVs induced in mock-treated WT cells could be expected to correlate with the aging-specific signature 1, which is dominated by CG>TG mutations at methylated CpGs.^11^ The correlation observed here is fairly weak, as CG>TG changes are overrepresented in human cancer samples to a far greater extent than in the experimental DT40 samples, where the WT, *BRCA1*^*+/−*^ and *BRCA2*^*+/−*^ samples all showed 11-fold overrepresentation (Figure 5d). The single *PCNA*^*K164R*^ sample had a greater proportion of CG>TG mutations owing to a reduction of other mutation types (Figures 2a and 5d), and indeed showed a better correlation with signature 1 (Figure 5a). SNV spectra from MMS-treated samples did not generally show good correlation with cancer mutation signatures apart from varied correlation with the broad-spectrum signature 8 with unknown aetiology (Figure 5a), but the MMS spectra correlated strongly with each other (Figure 5b). In conclusion, the mutation spectra measured in our controlled genetic model are in good agreement with correlative observational data obtained from BRCA1/2-defective human tumours.

### Prolonged DNA damage signalling in BRCA1 and BRCA2-defective cells

**Figure 6:**
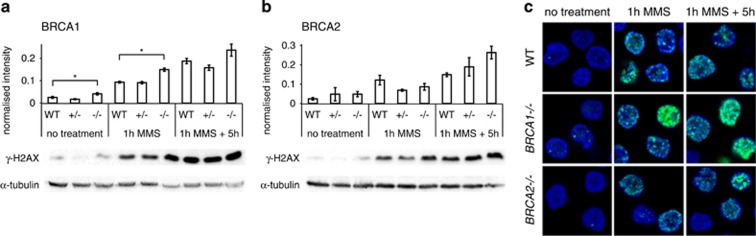
Markers of DNA damage response. (**a** and **b**) Western blots detecting γ-H2AX in WT cells as well as in *BRCA1* heterozygous (^+/−^) and homozygous (^*−/−*^) cells (**a**); and in *BRCA2* heterozygous (^+/−^) and homozygous (^*−/−*^) cells (**b**); subjected to no treatment or to 1 h treatment with 80 ppm MMS with or without a 5 h recovery period as indicated. Above the representative chemiluminescence images, mean values of normalized signal from *n*=3 to 4 experiments are shown. Error bars indicate s.e.m. Significant differences of values between WT and homozygous samples are indicated (paired *t*-test, *P<0.05). (**c**) Representative immunofluorescence images of γ-H2AX (green) and DNA (blue) in nuclei of WT, *BRCA1*^*−/−*^ and *BRCA2*^*−/−*^ cells subjected to the same treatments as in (**a** and **b**).

To better understand the potential mechanisms underlying the strong and near-identical mutagenic phenotypes of *BRCA1* and *BRCA2*, we asked if the mutant cell lines showed a higher level of markers of DNA damage. Phosphorylation of histone H2AX on serine 139 is one of the early markers of both double-strand breaks^36^ and replication fork stalling, such as that induced by thymidine arrest^37,38^ and is required for the recruitment of various repair factors including BRCA1.^39^ We found a low level of phosphorylated H2AX (γ-H2AX) in untreated cells (Figure 6a) with a significant increase in *BRCA1*^*−/−*^ versus WT cells (*P*=0.049, paired *t*-test). This difference was even more pronounced immediately after DNA-damaging treatment with MMS for 1 h (*P*=0.015). After a 5 h recovery period, the γ-H2AX signal increased further in the WT and *BRCA1*^*−/−*^ cells (Figure 6a). We saw a similar overall pattern of γ-H2AX levels in untreated and MMS-treated *BRCA2*^*−/−*^ cells, though here the differences to the WT were not significant (Figure 6b). The increased γ-H2AX levels following MMS treatment correlated with the appearance of a very large number of subnuclear γ-H2AX foci (Figure 6c), which were present in a proportion of cells consistent with the marking of stalled replication forks by γ-H2AX in S phase. The sustained and slightly increased γ-H2AX signal suggests a failure or delay of processing of stalled forks in *BRCA1*^*−/−*^ and *BRCA2*^*−/−*^ mutant cells.

## Discussion

In this study, we used isogenic cell lines as a model system to carefully test the mutagenic effect of *BRCA1* or *BRCA2* inactivation. In addition to finding an increased number of indels, we found a substantial increase in the number of spontaneously arising base substitutions that resembled a mutation signature associated with *BRCA1* and *BRCA2* mutant cancers. Our data support a role for the loss of *BRCA1/2* gene function in oncogenesis through increasing the rate of base substitution mutagenesis.

Several studies on *BRCA1* and *BRCA2* mutant tumour samples have focused on genomic scars derived from inaccurate break repair.^7, 8, 9^ Although *BRCA1/2* defect-associated SNV patterns have also been documented, cancer genomes give limited information on mutation rates. One reason for this is the lack of isogenic controls, as non-*BRCA1/2* mutant tumour samples have a range of different somatic mutations, and may even have affected *BRCA1/2* expression. In addition, a cancer genome is a snapshot of the genome of a cancer cell that started clonal expansion to give rise to the sampled part of the tumour, and the number of cell divisions and length of time between the *BRCA1/2* loss and the beginning of this expansion is impossible to tell. So whereas a higher median of SNV numbers has indeed been observed in *BRCA1/2*-defective breast cancer samples,^34^ the mutation rates are not known. In contrast, in a controlled system of simultaneously cultured isogenic WT and mutant cell clones we detected a massively higher spontaneous mutation rate in *BRCA1* or *BRCA2* mutant DT40 cells. Despite the ease of gene inactivation by homologous gene targeting, the similarity of HR rates in DT40 and cultured human cell lines has been demonstrated by, for example, similar sister chromatid exchange rates per chromosome,^40, 41^ and DT40 mutants have been used extensively for studying the genetics of HR.^42^ The similarity of the mutation patterns to signature 3 associated with *BRCA1/2* mutant cancers confirms the validity of the cell line model, and suggests that these cancers also have a higher mutation rate.

What could be the cause of the elevated mutation rate of *BRCA1* and *BRCA2* mutant cells? Note that although the SNV patterns in the *BRCA1* and *BRCA2* mutant cell lines were identical, there were both qualitative and quantitative differences in the arising indels, suggesting that SNVs and indels arise via different mechanisms. Deletions may mainly arise at collapsed replication forks, and the *BRCA2* deletion phenotype fully agrees with NHEJ acting on double-strand breaks unrepaired by HR. The milder *BRCA1* deletion phenotype suggests a decision point between the action of BRCA1 and BRCA2 in the process of fork collapse such that in the absence of BRCA2 mutagenic NHEJ is unavoidable, whereas in the absence of BRCA1 alternative DNA bypass mechanisms can still operate.

A likely source of SNVs and one-base indels is inaccurate DNA replication by translesion DNA polymerases. We obtained some evidence for this by observing a change in both the spontaneous and MMS-induced mutation spectrum in *PCNA*^*K164R*^ mutant cells, in which TLS is reduced and its mutagenic profile is altered.^31, 32^ This is not conclusive evidence, and the connection between *BRCA1/2* defects and TLS will need further investigation. Nevertheless, the identical MMS-induced SNV spectra suggest that the same error-prone TLS process is employed more frequently in *BRCA1/2* mutants than in WT cells. The increased use of TLS could be explained by the dependence of an error-free alternative bypass mechanism on *BRCA1/2*. This cannot be HR on a broken fork, as TLS cannot act after the damaged strand is cleaved. Template switch to the undamaged sister chromatid without fork collapse is a long-studied error-free third alternative bypass mechanism,^43^ with several possible variants that require RAD51 and other HR factors.^44^ Our best model, therefore, implicates the BRCA1 and BRCA2 proteins in template switching; in the absence of BRCA1/2 more frequent TLS would lead to the increased SNV rate. Both BRCA1 and BRCA2 have been shown to contribute to replication fork stabilisation independent of double-strand break repair^45, 46^ and this stabilisation through RAD51 loading could promote template switching, a scenario supported by the requirement for BRCA1 in promoting postreplicative gap repair and suppressing TLS at ultraviolet-induced lesions.^47^

Alternative explanations for increased mutagenesis in *BRCA1/2* cells may also be proposed by analogy to how Pol32-dependent error-prone DNA synthesis has been observed in the repair of broken forks by break-induced replication in *Saccharomyces cerevisiae.*^48^ However, the high number of γ-H2AX foci in MMS-treated cells are unlikely to primarily arise at distinct broken forks. Further, a mutagenic process operating only in *BRCA1/2* cells should alter the observed mutation spectra, which was not the case; and error-prone replication might be expected to produce several mutations within one event, but we did not observe a clustering of mutations.

The effect of BRCA1 and BRCA2 on carcinogenesis has mainly been considered in terms of the mutator hypothesis,^49^ with the inactivation of the genes destabilising the genome and leading to further gene mutations. Our discovery of high SNV mutation rates in *BRCA1/2* mutant cells lends further evidence to this theory. This also predicts that *BRCA1/2* mutations should be early ‘founder' mutations in cancer, which is supported by a few *BRCA1* cases in multiregion tumour sequencing.^50, 51^ The relative contribution of the increased SNV rates and the indel/rearrangement phenotype to the mutator function remains unknown. Considering that the SNV mutagenesis phenotypes of the two knockouts were identical, whereas there were several differences in the indel phenotypes, we propose that high base substitution rates are important to those clinical features of BRCA1 or BRCA2 mutant tumours that are similar, such as age of onset, tissue of origin. We found no significant difference between SNV or indel mutagenesis rates in wild-type versus *BRCA1*^*+/−*^ or *BRCA2*^*+/−*^ heterozygous cells, and detected too few large-scale rearrangements to draw conclusions. In contrast, impaired HR,^27^ defective suppression of replication fork collapse^25^ and an increase in chromosomal abnormalities^24^ have been observed in *BRCA1*^*+/−*^ human mammary epithelial cells. Thus carcinogenesis in *BRCA1/2* germline mutation carriers may take place in two hypothetical steps: the documented haploinsufficiency accelerates the inactivation of the second *BRCA1/2* allele via large-scale rearrangements, and the high SNV mutagenesis rate in the arising *BRCA1/2* homozygote leads to faster mutation of further cancer genes.

The various related and distinct functions of BRCA1 and BRCA2 have made it difficult to pinpoint the function of these gene products responsible for the closely related cancer phenotypes caused by their gene defects. Much research has focused on the connected roles of BRCA1 and BRCA2 in HR, suggesting that impaired HR in their absence leads to genome instability that accelerates tumour development. Our results have demonstrated an important additional aspect of the loss of BRCA1/2. The sevenfold elevated rate of base substitution mutations in both *BRCA1* and *BRCA2* mutant cells indicates a significant cause of genome instability. Considering the identical SNV phenotypes but distinct indel phenotypes of *BRCA1* and *BRCA2* mutant cell lines, a high SNV mutation rate may also be an important cause of the oncogenic effect of the loss of *BRCA1* or *BRCA2* function.

## Materials and methods

### Cell culture and drug treatments

The following DT40 cell lines were used: wild-type Clone18,^52^ *BRCA1*^*−/−*^ and *BRCA1*^*+/−*^ mutants,^19^ *BRCA2*^*−/−*^ and *BRCA2*^*+/−*^ (originally termed *BRCA2*^*-/con1*^),^20^ *PCNA*^*K164R/K164R*^ (referred to in the text as *PCNA*^*K164R*^).^33^ All gene mutations were authenticated using the whole-genome sequence data. Cells were grown at 37 °C under 5% CO_2_ in Roswell Park Memorial Institute-1640 medium supplemented with 7% fetal bovine serum and 3% chicken serum. MMS (Sigma-Aldrich, St Louis, MO, USA) or mock treatments were performed on one million cells for 1 h. Single cell clones were isolated and expanded to two million cells prior to genomic DNA preparation using the Gentra Puregene Cell Kit (Qiagen, Hilden, Germany). MMS sensitivity was measured by counting surviving cell colonies after plating treated cells in medium containing 1% methylcellulose.

### Western blotting and immunofluorescence

Whole-cell extracts were fractionated by sodium dodecyl sulphate–olyacrylamide gel eletrophoresis, transferred to polyvinylidene difluoride membranes and incubated with primary antibodies against γH2AX (Millipore, Merck, Darmstadt, Germany, 05-636, 1:4000) or alpha-tubulin (Sigma-Aldrich T6199, 1:2000); followed by secondary anti-mouse (Sigma-Aldrich A9044, 1:20000) or anti-rabbit (Sigma-Aldrich A0545, 1:20000) antibodies. Blots were developed with the ECL system and imaged with a Chemidoc MP instrument (Bio-Rad Laboratories, Hercules, CA, USA). Band intensities were normalized to alpha-tubulin detected on the same membrane. Before the averaging of measurements, the sum of signals on each membrane was normalized to one.

For immunofluorescence analysis, cells were pelleted onto poly-l-lysine-coated coverslips and fixed with 4% paraformaldehyde. After blocking with 0.1% Tween 20 and 0.02% sodium dodecyl sulphate in phosphate-buffered saline, the samples were sequentially incubated with anti-γH2AX antibody (Millipore 05-636, 1:1000) and Alexa Fluor 488 anti-mouse secondary antibody (Thermo Fisher Scientific, Waltham, MA, USA, A-11029, 1:1000) for 1 h each at 37 °C followed by Hoechst 33342 (Thermo Fisher Scientific H3570, 1:10000) at room temperature for 10 min. The fluorescent signal was detected with a Zeiss LSM 710 confocal microscope.

### Whole-genome sequencing, mutation calling and data analysis

Library preparation used the TruSeq DNA Nano Library Preparation Kit (Illumina, San Diego, CA, USA) or the NEBNext Ultra DNA Library Prep Kit for Illumina (New England Biolabs, Ipswich, MA, USA). Sequencing was done on Illumina HiSeq 2500 (2 × 150 bp paired end (PE) format, three samples), Illumina HiSeq 2500 v4 (2 × 125 bp PE, 21 samples) and Illumina HiSeq X Ten instruments (2 × 150 bp PE, six samples). Library preparation and DNA sequencing was done at the Research Technology Support Facility of Michigan State University, USA, and at Novogene, Beijing, China. We chose to sequence three samples per treatment to be able to detect sample variance. All data sets from successfully sequenced samples were used for subsequent analysis.

The reads were aligned to the chicken (Gallus gallus) reference sequence Galgal4.73 as described.^53^ Duplicate reads were removed using samblaster.^54^ The aligned reads were realigned with GATK IndelRealigner.^55^

Independently arising SNVs and short indels were identified using the IsoMut method developed for multiple isogenic samples.^22^ In brief, after applying a base quality filter of 30, data from all samples were compared at each genomic position, and filtered using optimized parameters of minimum mutated allele frequency (0.2), minimum coverage of the mutated sample (5) and minimum reference allele frequency of all the other samples (0.93), and also filtered using a probability-based quality score calculated from the mutated sample and one other sample with the lowest reference allele frequency (Supplementary file S6, Supplementary table S1). The IsoMut code is available for unrestricted download.^56^ Structural variations were detected using the CREST algorithm.^57^

Ninety-six-triplet signatures^11^ were generated after pooling samples of the same genotype and treatment. DT40 triplet signatures were adjusted with the ratio of each triplet occurrence in the human and chicken genome and compared with the 30 human cancer triplet signatures^58^ using Pearson correlation coefficient. Two-sided *t*-tests were used for statistical comparisons of mutation numbers with no adjustments for multiple comparisons, Fisher's exact test was used to compare categorized mutations, and the non-parametric Kolmogorov–Smirnov test was used to compare the size distribution of deletions.

Raw sequence data has been deposited with the European Nucleotide Archive under study accession number ERP015181.

## Figures and Tables

**Table 1:** Number of SNV and short insertion/deletion mutations in the sequenced samples

*Treatment*	n	*SNV mean±s.d.*	*Insertion mean±s.d.*	*Deletion mean±s.d.*
*WT*				
Starting clone	1	4	0	0
Mock	3	72±5	4.7±1.5	1.7±0.6
MMS	3	1489±620	5.5±2.5	6.0±2.1

*BRCA1*^*+/−*^				
Starting clone	1	5	0	0
Mock	2	63±14	4.0±1.3	2.5±1.5
MMS	3	1582±840	3.0±1.7	9.3±3.5

*BRCA1*^*−/−*^				
Starting clone	1	1	1	0
Mock	3	562±75	8.0±1.0	12.7±1.2
MMS	3	2414±201	7.3±2.1	24.7±6.5

*BRCA2*^*+/−*^				
Starting clone	1	2	0	1
Mock	3	79±13	2.7±1.2	2.0±2.0
MMS	2	1629±88	3.5±2.1	9.0±4.2

*BRCA2*^*−/−*^				
Starting clone	1	2	0	0
Mock	3	511±21	10.3±3.1	33.0±5.0
MMS	3	2986±324	11.7±4.2	40.3±2.1
				
*PCNA*^*K164R*^				
Starting clone	1	1	0	0
Mock	1	43	5	7
MMS	1	2286	3	6

Abbreviations: MMS, methyl methanesulfonate; SNV, single-nucleotide variation; WT, wild type. Independent mutations in starting clones represent false positives of the mutation detection.
