# Erratum: Loss of BRCA1 or BRCA2 markedly increases the rate of base substitution mutagenesis and has distinct effects on genomic deletions

**DOI:** 10.1038/onc.2017.213

**Published:** 2017-06-26

**Authors:** J Zámborszky, B Szikriszt, J Z Gervai, O Pipek, Á Póti, M Krzystanek, D Ribli, J M Szalai-Gindl, I Csabai, Z Szallasi, C Swanton, A L Richardson, D Szüts

**Keywords:** Mutagenesis, Oncogenes, Cancer genetics

**Correction to:** *Oncogene* (2017) **36**, 746–755; doi:10.1038/onc.2016.243; published online 25 July 2016

In Figure 2c, the label above the middle panel in this paper was published incorrectly. The correct label should read BRCA1−/− instead of BRCA2−/−.

The corrected figure 2 is below.

**Figure 2:**
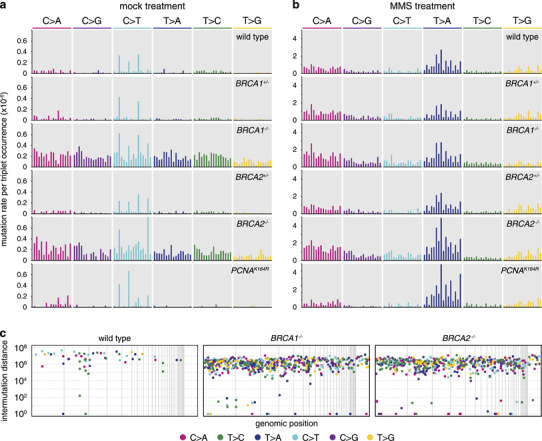
Triplet mutation spectrum and genomic distribution of SNVs. Triplet mutation spectra of the mock treatment (**a**) or MMS treatment (**b**) of the indicated cell lines. Each mutation class, as indicated at the top of the panel, is separated into 16 categories based on the identity of the preceding and following nucleotide. The mutation rate at each triplet was obtained by dividing the number of observed mutations with the number of occurrences of that particular triplet in the chicken genome. The sequence of triplets is shown on expanded Supplementary figures S2-S5; the four C>T peaks in mock-treated samples represent NCG>NTG mutations. (**c**) In mock-treated clones of the indicated genotypes, the distance of each SNV mutation from the previous SNV on the same chromosome is plotted against the genomic position of the mutation. Thin vertical lines indicate chromosome boundaries. Chromosomes are shown in numerical order, chromosome Z is shown last on the right. The colour of each dot illustrates the type of mutation according to the key at the bottom of the panel. One sequenced clone of each cell line is shown.

The publishers wish to apologise for any inconvenience caused.

