# Supplementary figures and images for: Loss of BRCA1 or BRCA2 markedly increases the rate of base substitution mutagenesis and has distinct effects on genomic deletions
Source: Oncogene. 2016 Jul 25;36(6):746–55. doi: 10.1038/onc.2016.243 (PMC5096687; doi:10.1038/onc.2016.243)

An outline of the design of the mutagenesis experiment with the timings indicated.

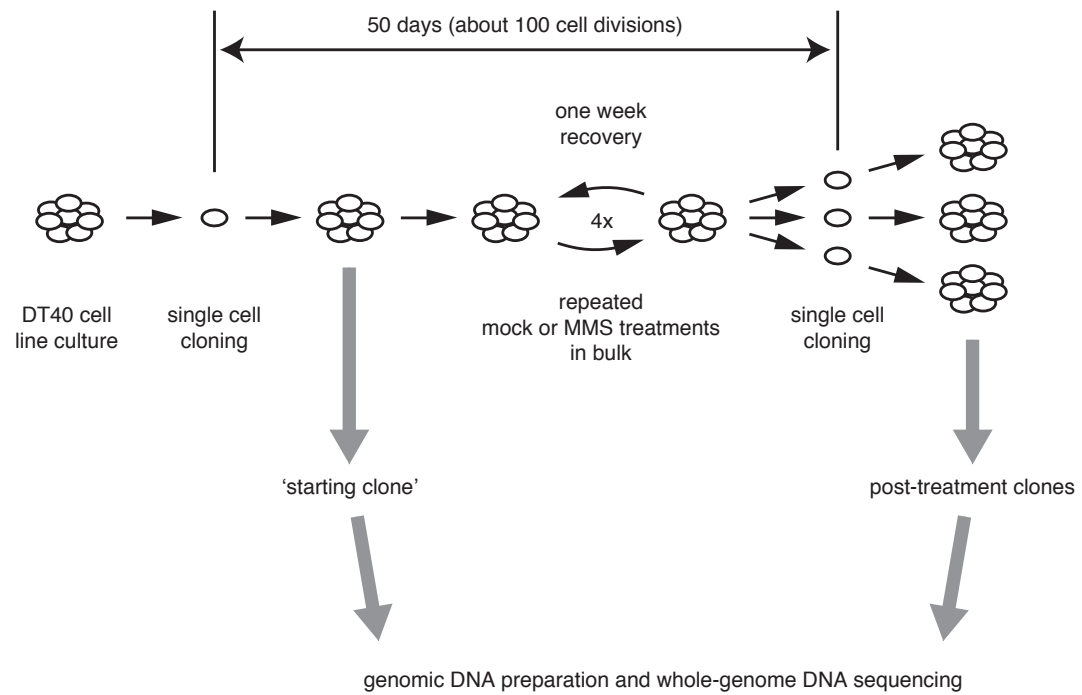

Supplement: Supplementary Figure 1 [file onc2016243x1.pdf]
