# Supplementary material for: Loss of BRCA1 or BRCA2 markedly increases the rate of base substitution mutagenesis and has distinct effects on genomic deletions
Source: Oncogene. 2016 Jul 25;36(6):746–55. doi: 10.1038/onc.2016.243 (PMC5096687; doi:10.1038/onc.2016.243)

Figure S2

Mock treatment induced SNV mutations in the context of the neighbouring bases, normalised to the genomic frequency of base triplets.

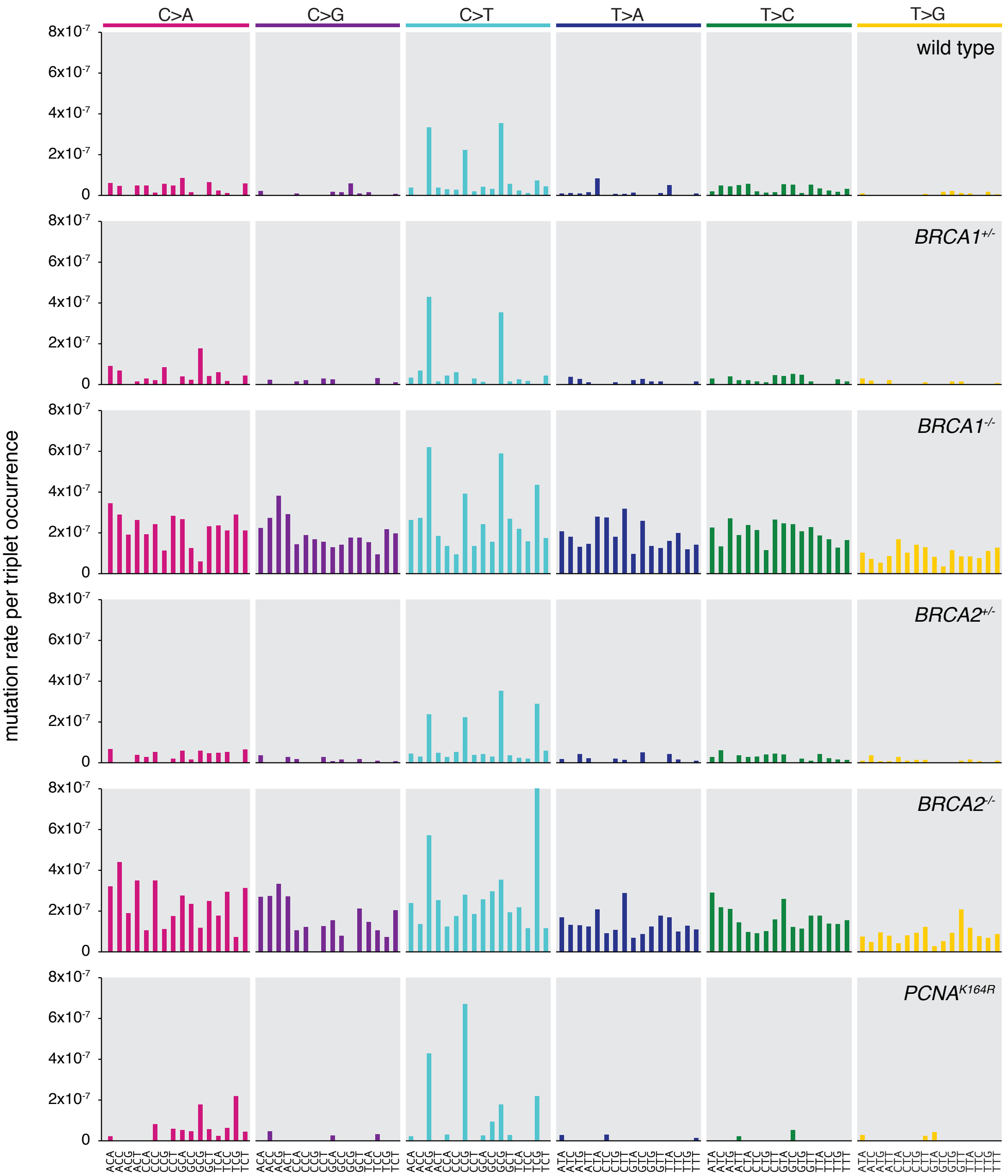

Figure S3

MMS treatment induced SNV mutations in the context of the neighbouring bases, normalised to the genomic frequency of base triplets.

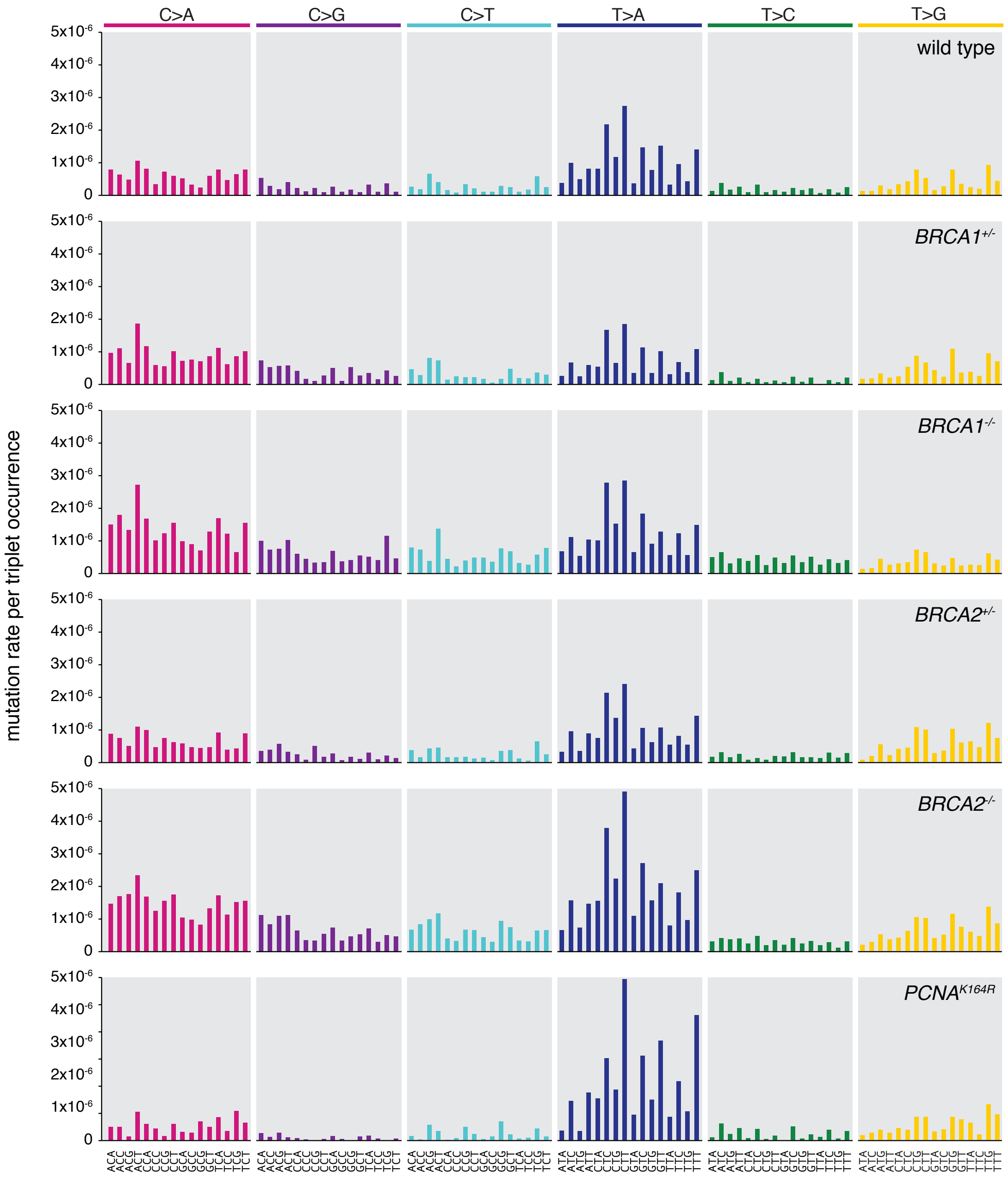

Figure S4

Mock treatment induced SNV mutations in the context of the neighbouring bases, not normalised to the genomic frequency of base triplets.

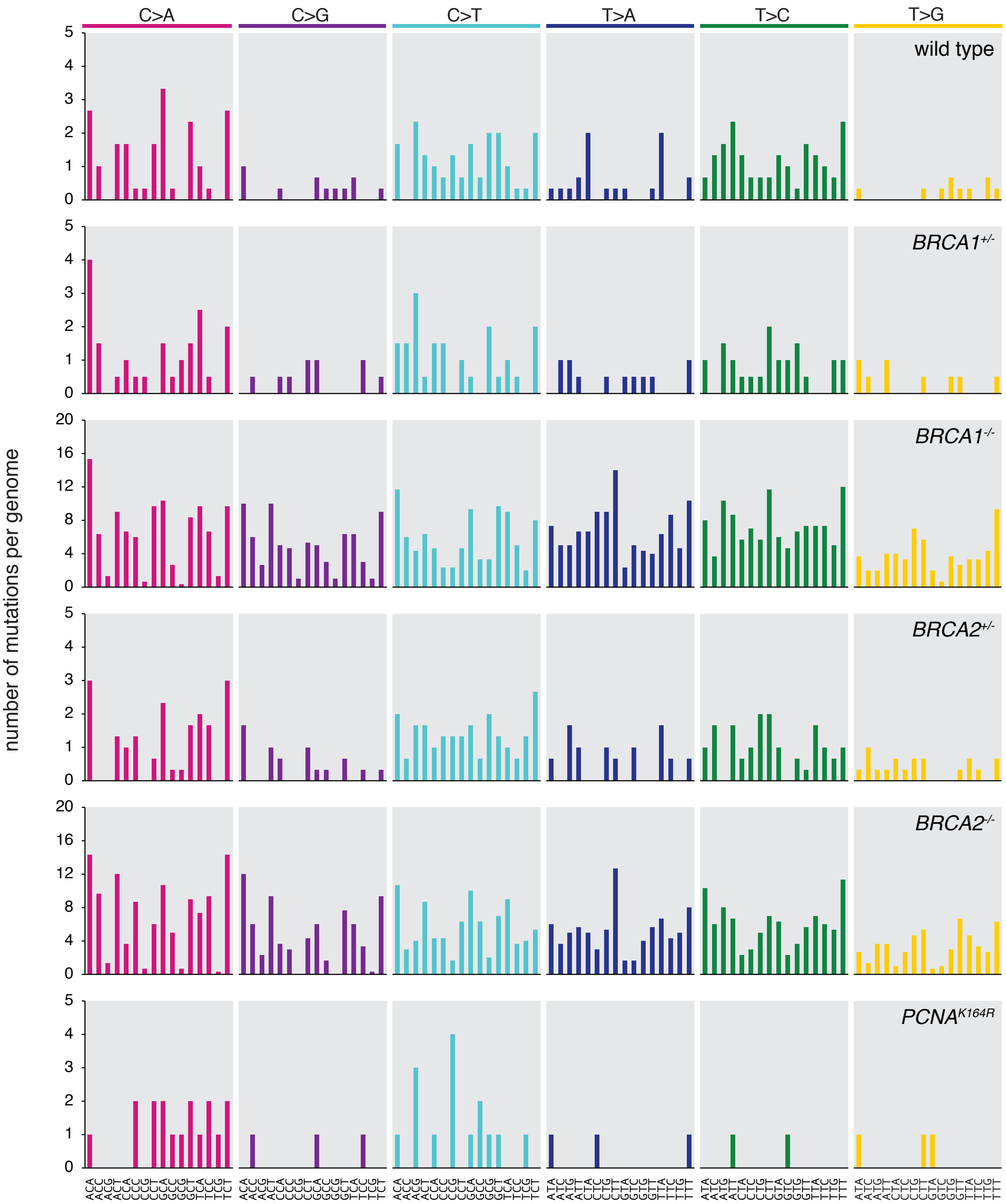

Figure S5

MMS treatment induced SNV mutations in the context of the neighbouring bases, not normalised to the genomic frequency of base triplets.

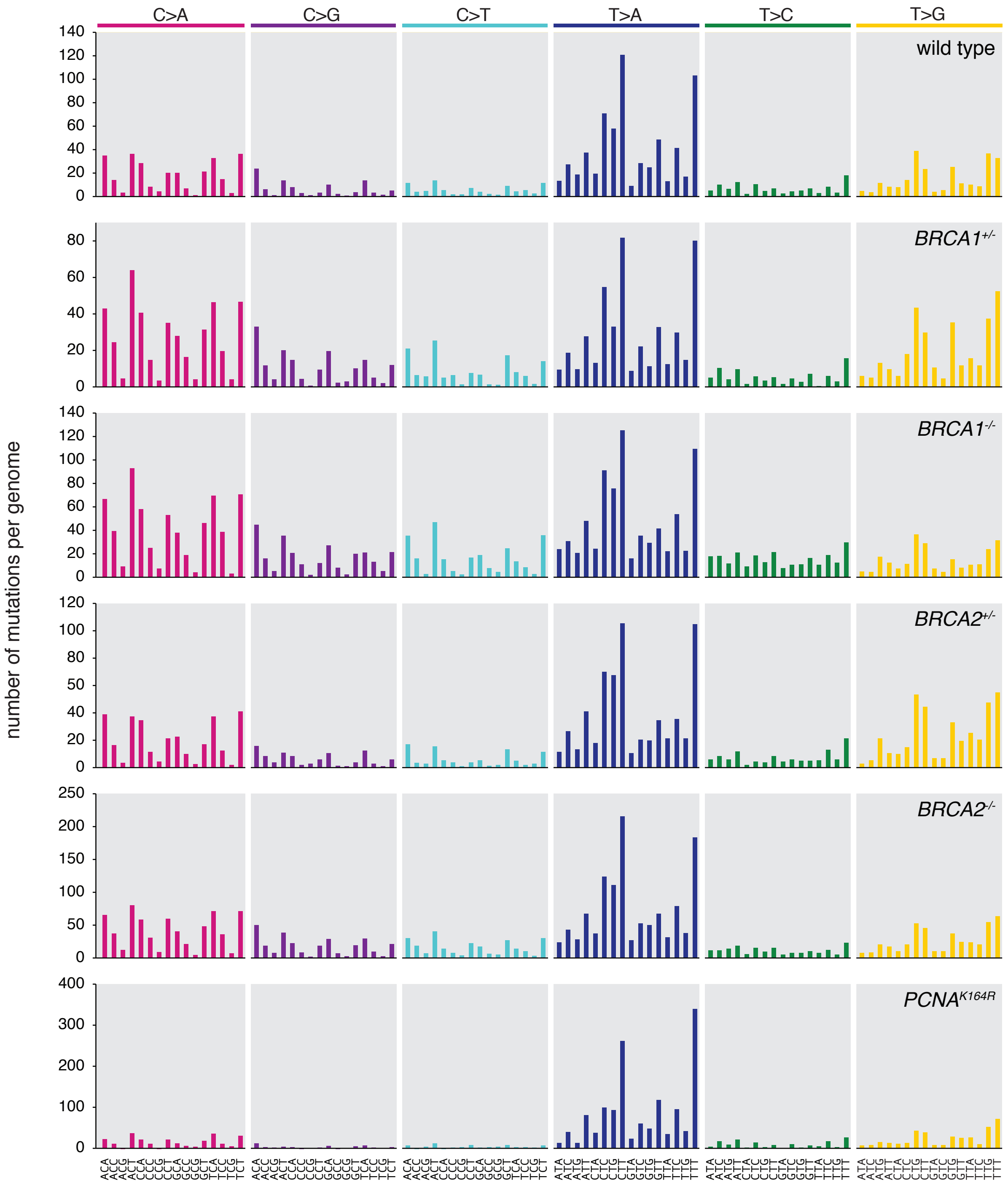

Supplement: Supplementary Figure 2 [file onc2016243x2.pdf]
