# Supplementary material for: Loss of BRCA1 or BRCA2 markedly increases the rate of base substitution mutagenesis and has distinct effects on genomic deletions
Source: Oncogene. 2016 Jul 25;36(6):746–55. doi: 10.1038/onc.2016.243 (PMC5096687; doi:10.1038/onc.2016.243)
Supplement: Supplementary Information [file onc2016243x3.pdf]

Supplementary file S6 ¶

TITLE

Loss of BRCA1 or BRCA2 markedly increases the rate of base substitution mutagenesis and has distinct effects on genomic deletions

AUTHORS

Judit Zámborszky, Bernadett Szikriszt, Judit Z. Gervai, Orsolya Pipek, Ádám Póti, Marcin Krzystanek, Dezső Ribli, János M. Szalai-Gindl, István Csabai, Zoltán Szallasi, Charles Swanton, Andrea L. Richardson, Dávid Szüts

Overview

Unique mutation detection in samples with IsoMut (https://github.com/riblidezso/isomut (https://github.com/riblidezso/isomut)).

Running IsoMut on all samples, with close to default parameters, and then optimize a score cutoff in the output.

I have tuned the cutoff in mutation quality score (score), using the control samples available. In these samples no unique, treatment-induced mutations should be present, thus the score can be tuned by minimizing the number of detected mutations in these samples, while maintaining satisfyingly high numbers in samples that underwent mutagenic treatments. The tuning procedure can be individually carried out for SNVs, insertions and deletions, achieving optimized results for all types of mutations.

The value of the score is related to the probability of false positive mutation. The score is calculated as the negative logarithm of Fisher's exact test p value on the the two 'noisiest' samples. Thus a higher score value means more confident mutation call. Please note that the score itself has no clear physical probabilistic interpretation, but is proved to be efficient for this purpose.

The control samples are either starting clones, or identical DNA pairs, therefore they should contain no unique mutations.

Repoducibilty, Notes

This notebook contains everything (excluding the data bam files) to run IsoMut on the samples described in the article. After downloading the data, and slight modifications, the analysis can be easily repeated on any computer.

Please note that we use the Slurm Workload Manager (http://slurm.schedmd.com/ (http://slurm.schedmd.com/)). Whenever a different workload manager is preferred, the notebook should be adjusted accordingly. The other parts of the code can be used without significant modifications.

Please note that analyzing (even reading) a terabyte data takes some time. On our 12 core computer the analysis takes 16 hours (actually i'm only using 8 because disk IO is the limiting factor anyway). Using only 1-2 cores or an old disk can make it much slower.

Please note that the notebook contains shell and python commands combined.

Steps:

- Download the software from github
- Compile it
- Modify the example script (input/output directories, sample info) and save it
- Submit job to queue using the preferred workload manager
- Analyze results

Preparations

Prepare the application

- download it, compile it make directory for output

In [1]:
```
%%bash

# Download from git
# git clone https://github.com/riblidezso/isomut.git
cd isomut

# compile
gcc -c -O3 isomut_lib.c fisher.c  -W -Wall
gcc -O3 -o isomut isomut.c isomut_lib.o  fisher.o -lm -W -Wall
```

In [2]:
```
import os
os.chdir('isomut/')
```

Modify example script ans save it

I have changed: sample names and input,output directory

In [3]:
```
%%writefile brca_article_samples.py
#!/usr/bin/env python
#################################################
# importing the wrapper
#################################################
#add path for isomput_parallel.py if its not here
import sys,os,subprocess
sys.path.append(os.getcwd())
```

```
#load the parallel wraooer function
from isomut_wrappers import run_isomut_with_pp

#################################################
# defining administrative parameters
#################################################
#using parameter dictionary, beacause there are awful lot of parame
ters
params=dict()
#minimum number of blocks to run
# usually there will be 10-20 more blocks
params['n_min_block']=100
#number of concurrent processes to run
params['n_conc_blocks']=8
#genome
params['ref_fasta']="/home/ribli/input/index/gallus/Gallus_gallus.G
algal4.74.dna.toplevel.fa"
#input dir output dir
params['input_dir']='/nagyvinyok/adat83/sotejedlik/ribli/dt40/bam_l
inks_2/'
params['output_dir']='output/'
#the bam files used
samples=['DS014','DS026','DS027','DS051','DS052','DS053','DS054','D
S055','DS056',

'DS059','DS060','DS061','DS066','DS067','DS068','DS103','DS104','DS
108',

'DS109','DS110','DS111','DS115','DS116','DS117','DS118','DS119','DS
120',

'DS121','DS125','DS126','DS141','DS142','DS143','DS144','DS151','DS
152',
         'DS153','DS154','DS155','DS156']
params['bam_filenames']=[sample+'_RMdup_picard_realign.bam' for sam
ple in samples ]

#limit chromosomes (for references with many scaffolds)
# just comment/delete this line if you want to analyze all contigs
in the ref genome
params['chromosomes']=map(str,range(1,29))+ ['32','W','Z']

#################################################
# defining mutation calling parameters
#    default values here ...
#################################################
params['min_sample_freq']=0.2
params['min_other_ref_freq']=0.93
params['cov_limit']=5
params['base_quality_limit']=30
params['min_gap_dist_snv']=0
params['min_gap_dist_indel']=20

#################################################
# and finally run it
#################################################
run_isomut_with_pp(params)
```

```
Overwriting brca_article_samples.py
```

Submit job to queue

In [4]:
```
%%bash
sbatch -c 24 --mem 15G -C jimgray83 brca_article_samples.py
```

Analyze results

Load python modules used

In [5]:
```
import pandas as pd
import numpy as np
import matplotlib.pyplot as plt
%matplotlib inline
```

Just dump the output into one file now, (this is because old output was in one file and the functions below were written for that)

In [6]:
```
%%bash

head -n1 output/all_SNVs.isomut    > all_output.csv
cat output/all_SNVs.isomut | grep -v sample >> all_output.csv
cat output/all_indels.isomut | grep -v sample >> all_output.csv
```

Load data

In [7]:
```
output=pd.read_csv('all_output.csv',sep='\t',header=0)
```

Plot the number of mutations found in each sample depending on the score threshold

In [8]:
```
##define sample groups
N=40
control_idx=[0,1,12,17,24,30]
not_control_idx=set(range(N))
for i in control_idx:
    not_control_idx.remove(i)
not_control_idx=sorted(not_control_idx)

def plot_tuning_curve(output,ymax):
    #set cols
    cols=['lightgreen' for i in xrange(N)]
    for i in control_idx:
        cols[i]='dodgerblue'

    fig,ax=plt.subplots()
    fig.set_size_inches(12,9)
    for i,col in zip(range(N),cols):
        score=output[(output['#sample_idx']==i)].sort_values(['scor
e'])['score']
        ax.plot(score,len(score)-np.arange(len(score)),c=col,lw=4,l
abel='')
    ax.set_xlabel(r'$\log_{10}(p) $ threshold',fontsize=16)
    ax.set_ylabel(r'Mutations found',fontsize=16)
    ax.set_ylim(1,ymax)
    ax.set_xlim(0,4)
    ax.grid()


    fig,ax=plt.subplots()
    fig.set_size_inches(12,9)
    for i,col in zip(range(N),cols):
        score=output[(output['#sample_idx']==i)].sort_values(['scor
e'])['score']
        ax.plot(score,len(score)-np.arange(len(score)),c=col,lw=4,l
abel='')

    ax.set_xlabel(r'$\log_{10}(p) $ threshold',fontsize=16)
    ax.set_ylabel(r'Mutations found',fontsize=16)
    ax.set_ylim(1,ymax)
    ax.set_xlim(0,4)
    ax.set_yscale('log')
    ax.grid()
```

Plot SNVs

- With score >~ 2.5 there are fewer than 10 mutations in the control samples, while the treated samples still have thousands of mutations. This shows, that IsoMut worked

In [9]:
```
plot_tuning_curve(output[output['type']=='SNV'],4e3)
```

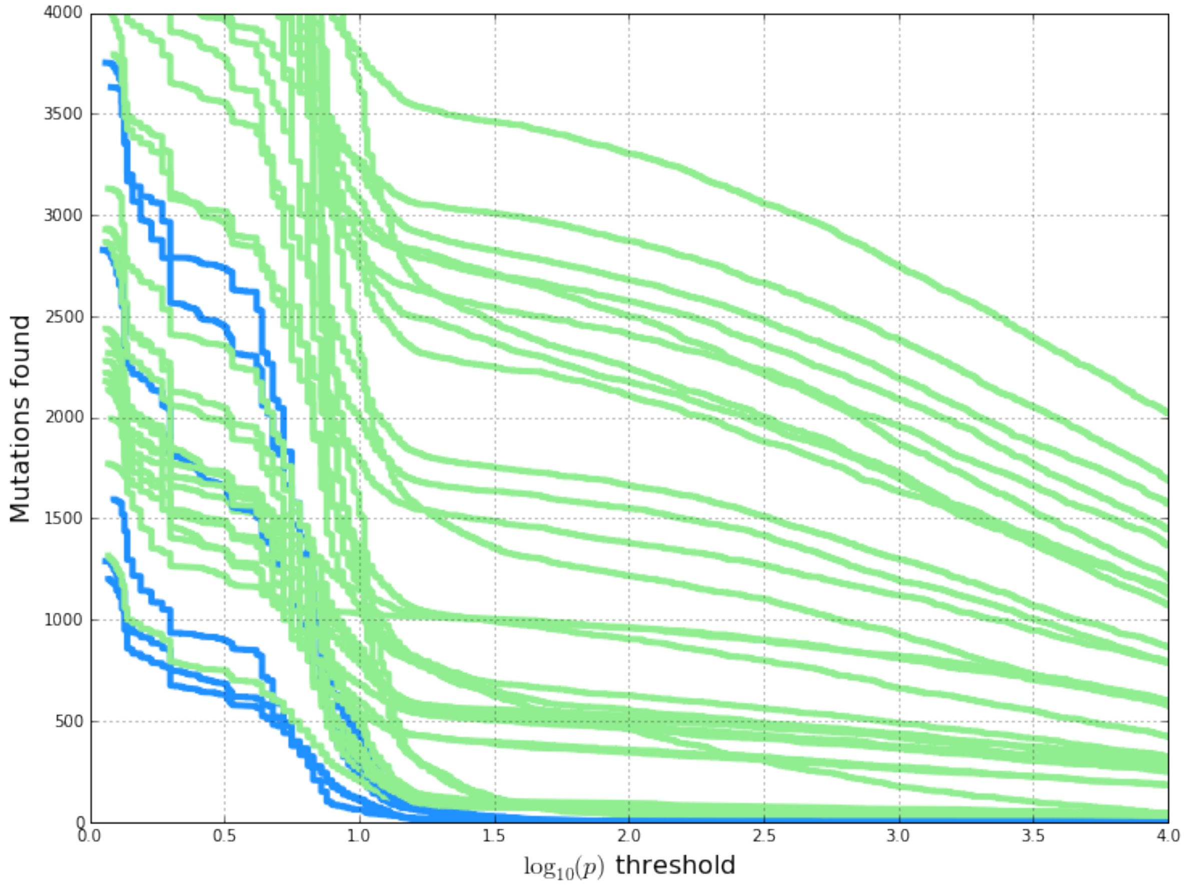

4000
3500
3000
2500
2000
1500
1000
500
0
Mutations found
0.0
0.5
1.0
1.5
2.0
2.5
3.0
3.5
4.0
$\log_{10}(p)$ threshold

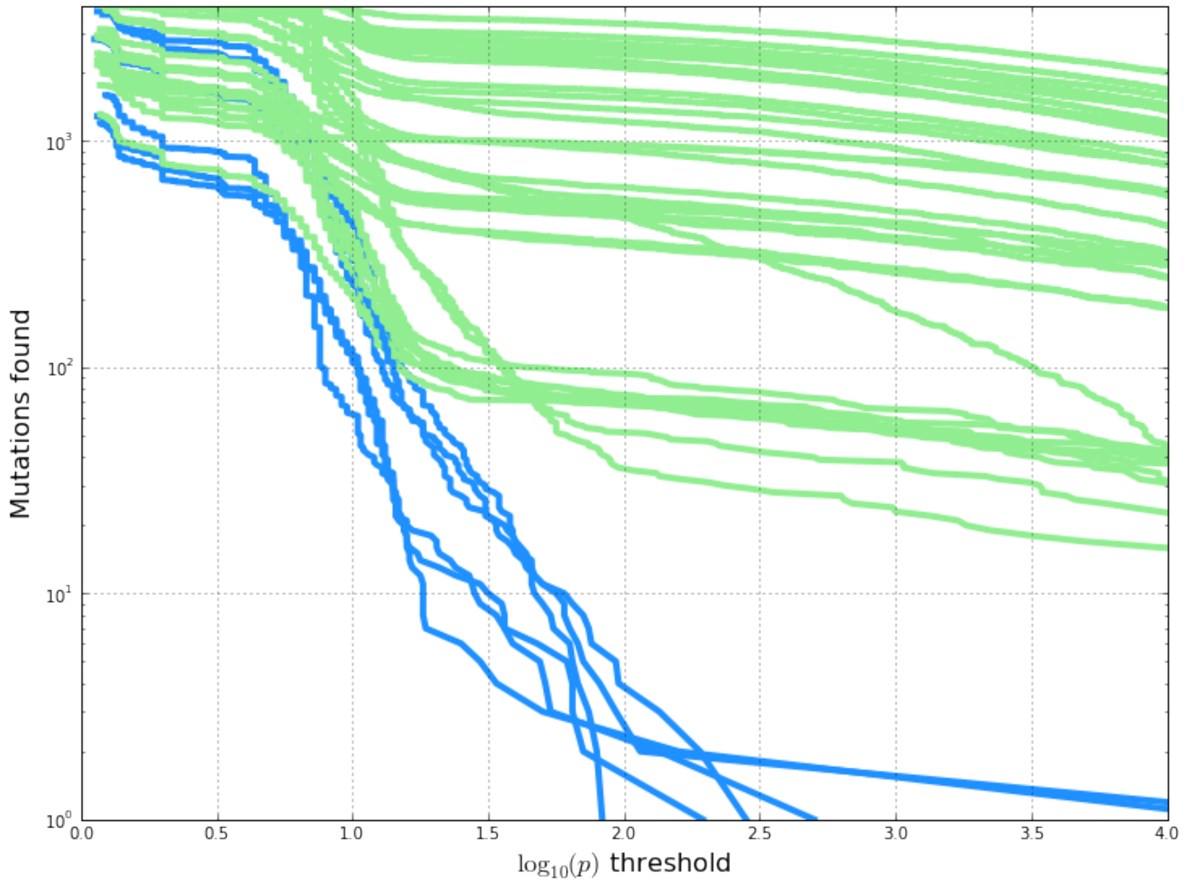

10^3
10^2
10^1
10^0
Mutations found
0.0
0.5
1.0
1.5
2.0
2.5
3.0
3.5
4.0
$\log_{10}(p)$ threshold

Plot insertions

- With score >~ 1.5 there are fewer almost no mutations in the control samples, while the treated samples still have around a hundred of mutations. This shows, that IsoMut worked

In [10]:
```
plot_tuning_curve(output[output['type']=='INS'],40)
```

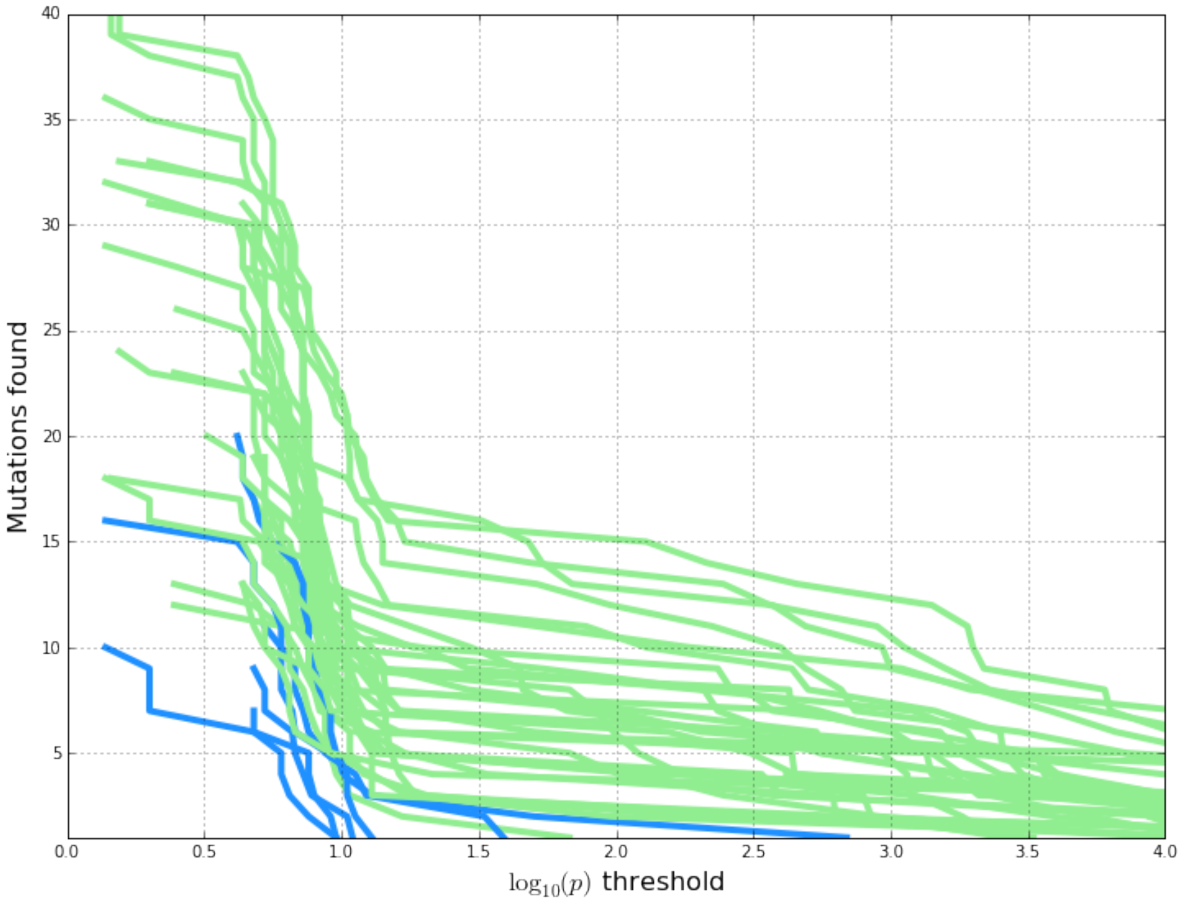

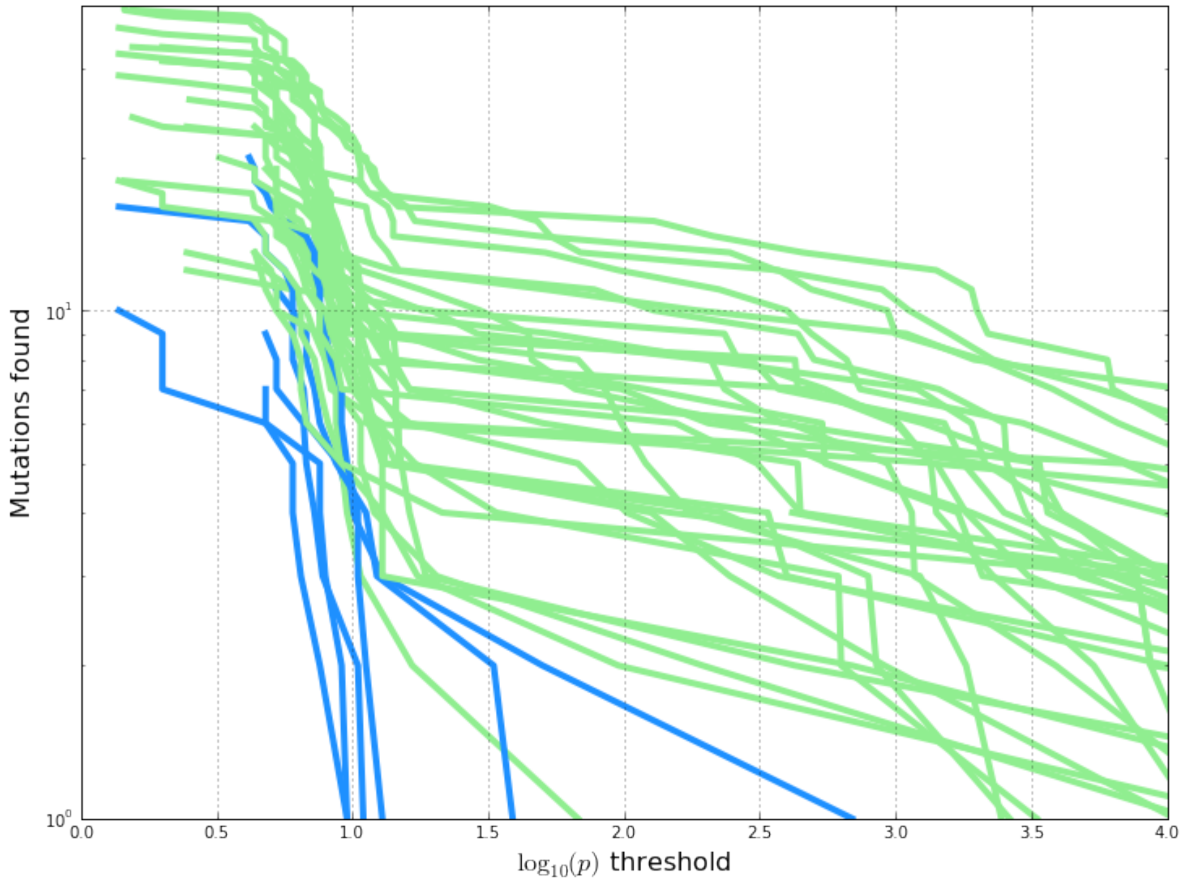

Plot deletions

- With score >~ 1.5 there are fewer almost no mutations in the control samples, while the treated samples still have around a hundred of mutations. This shows, that IsoMut worked

In [11]:
```
plot_tuning_curve(output[output['type']=='DEL'],70)
```

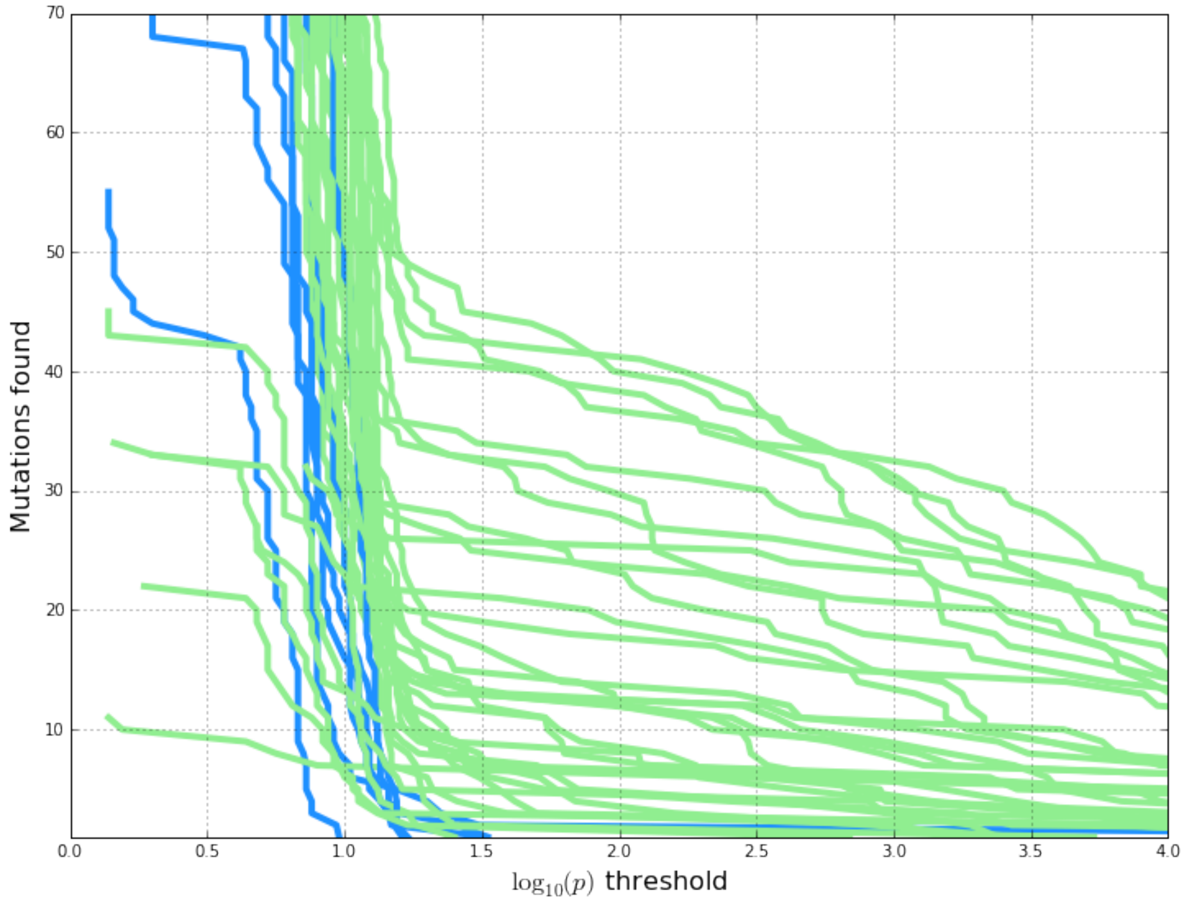

70
60
50
40
30
20
10
Mutations found
0.0
0.5
1.0
1.5
2.0
2.5
3.0
3.5
4.0
$\log_{10}(p)$ threshold

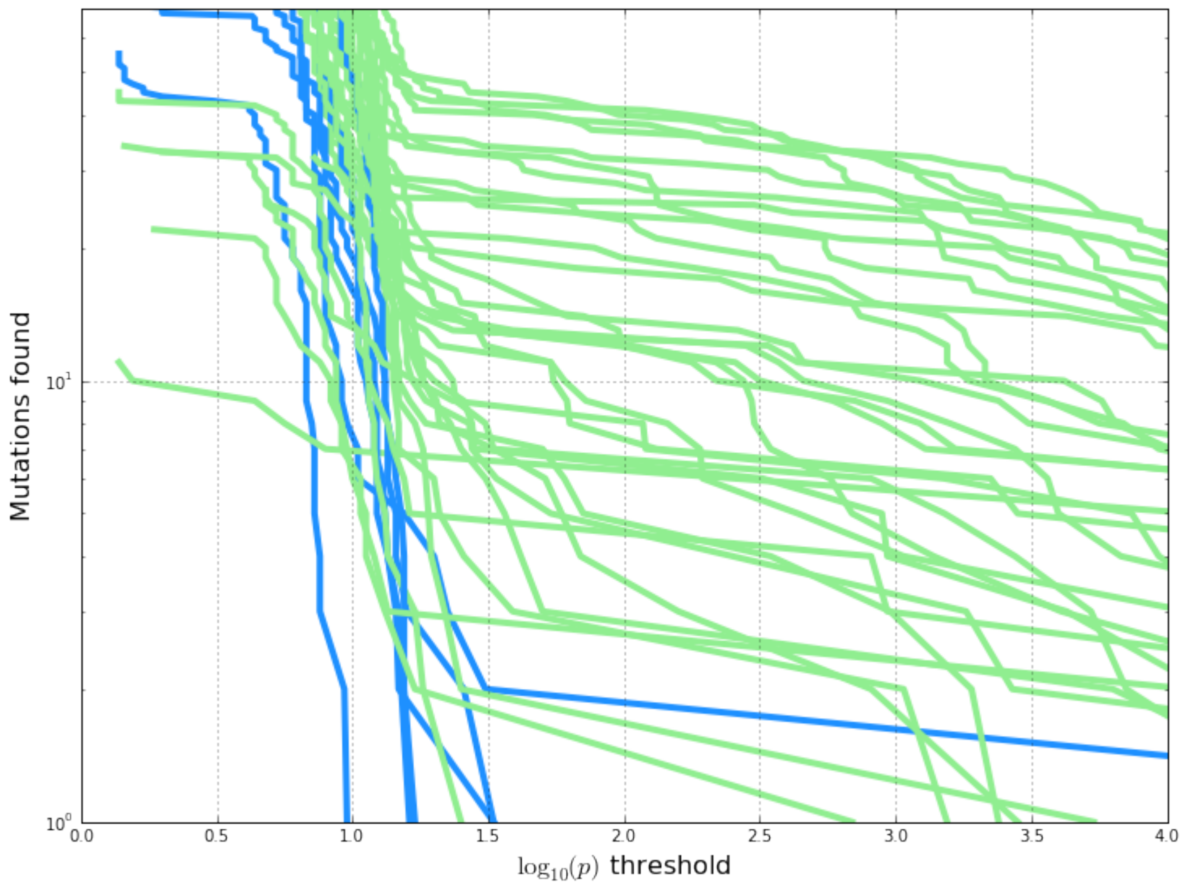

10^1
10^0
Mutations found
0.0
0.5
1.0
1.5
2.0
2.5
3.0
3.5
4.0
$\log_{10}(p)$ threshold

Plotting a quasi-ROC curve for score optimization

- False positive rate: the ratio of mutations found in control samples to the length of the genome
- For a fast and convenient optimization process, true positives were estimated as the mutations found in all treated samples. Some of these may in fact be false positives, but as the mutation rate in treated samples are higher by a factor of 100-1000 than in control samples, this estimation still gives reasonable results.
- The ROC curves below show how the change in the selected score threshold affects the number of false and true positive results. For our purposes, very low false positive rates were necessary, thus we chose fairly strict filtering values to achieve this.

In [12]:
```
def plot_roc(output,score0,score_sel):

    fig,ax=plt.subplots()
    fig.set_size_inches(12,9)
    fp, tp = [0  for i in xrange(100) ],[0  for i in xrange(100) ]
    for score_lim,j in zip(np.linspace(score0,10,100),range(100)):
        muts=[]
        for i in xrange(N):
            filt_idx =  output['#sample_idx'] ==  i
            filt_idx = filt_idx & ((output['score']>score_lim))
            muts.append(len(output[filt_idx]))
        muts=np.array(muts)
        fp[j] ,tp[j]=1e-3*np.mean(muts[control_idx]),1e-3*np.mean(m
uts[not_control_idx])
    ax.step(fp,tp,c='magenta',lw=4,label='quasy ROC, scannning the
tuning parameter')

    muts=[]
    for i in xrange(N):
        filt_idx =  output['#sample_idx'] ==  i
        filt_idx = filt_idx & ((output['score']>score_sel))
        muts.append(len(output[filt_idx]))
    muts=np.array(muts)

    ax.plot(1e-3*np.mean(muts[control_idx]),1e-3*np.mean(muts[not_c
ontrol_idx]),
            'o',mec='dodgerblue',mfc='dodgerblue',ms=15,mew=3,label
='selected parameter')
    ax.legend(fancybox=True,loc='lower right',fontsize=16)

    ax.set_ylim(ymin=0)
    ax.set_xlim(xmin=-0.0001)
    ax.set_xlabel('false positive rate 1/Mbp ',fontsize=16)
    dump=ax.set_ylabel('mutation rate  1/Mbp ',fontsize=16)
```

SNVs

- We have chosen a fairly weak threshold

In [13]:
```
plot_roc(output[output['type']=='SNV'],score0=1.5,score_sel=1.9)
```

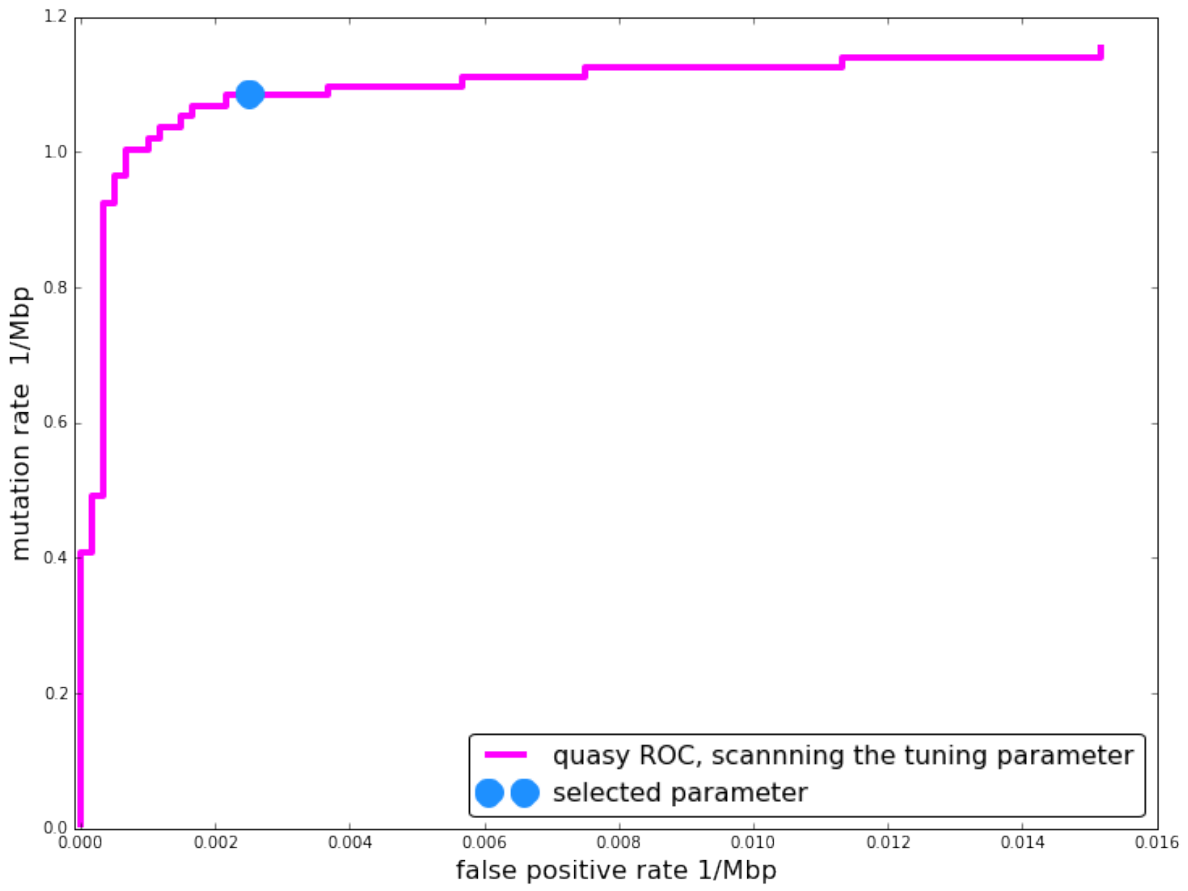

INS

- please note, that the score threshold for insertions/deletions is very different than score threshold for SNVs, because the insertions have different noise frequencies, and because of alignement errors, they have different frequencies even when they are real.

In [14]:
```
plot_roc(output[output['type']=='INS'],score0=1,score_sel=1.7)
```

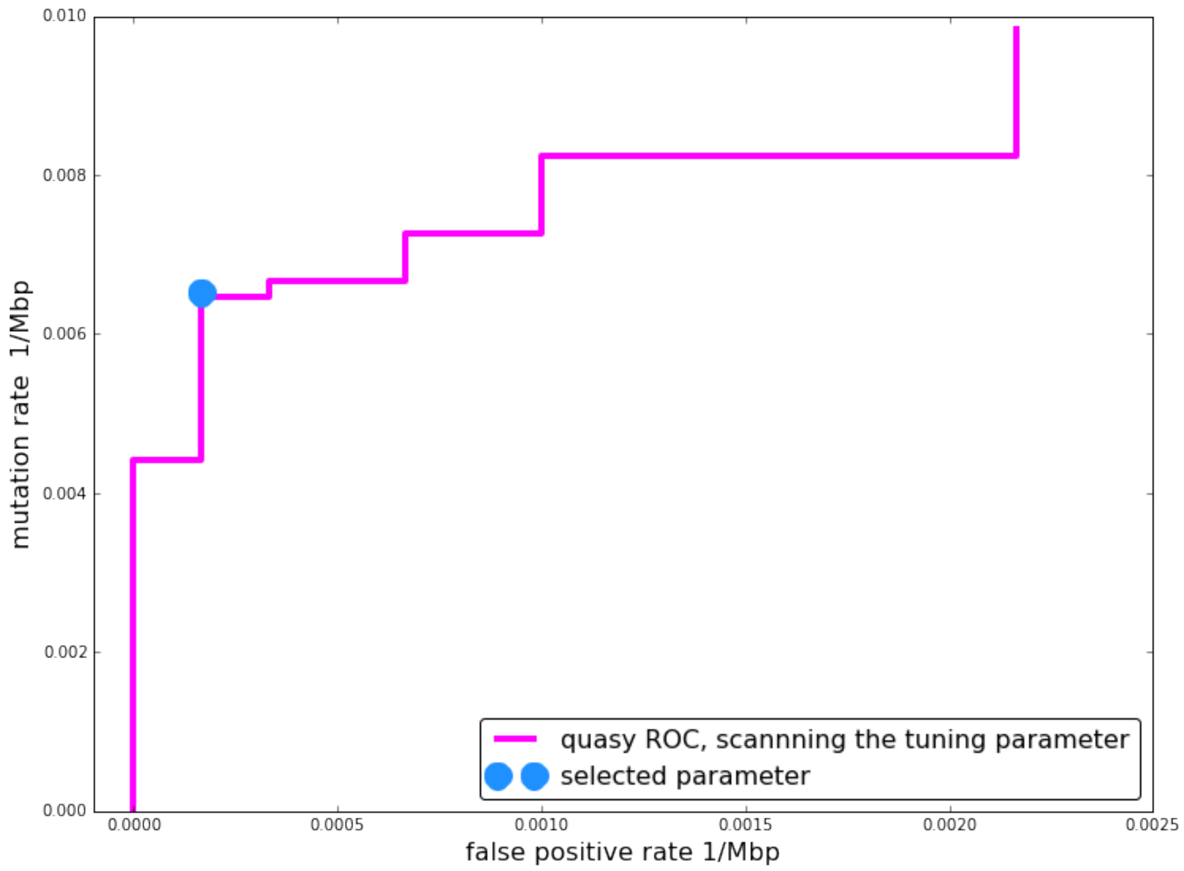

DEL

```
In [15]: plot_roc(output[output['type']=='DEL'],score0=1,score_sel=1.8)
```

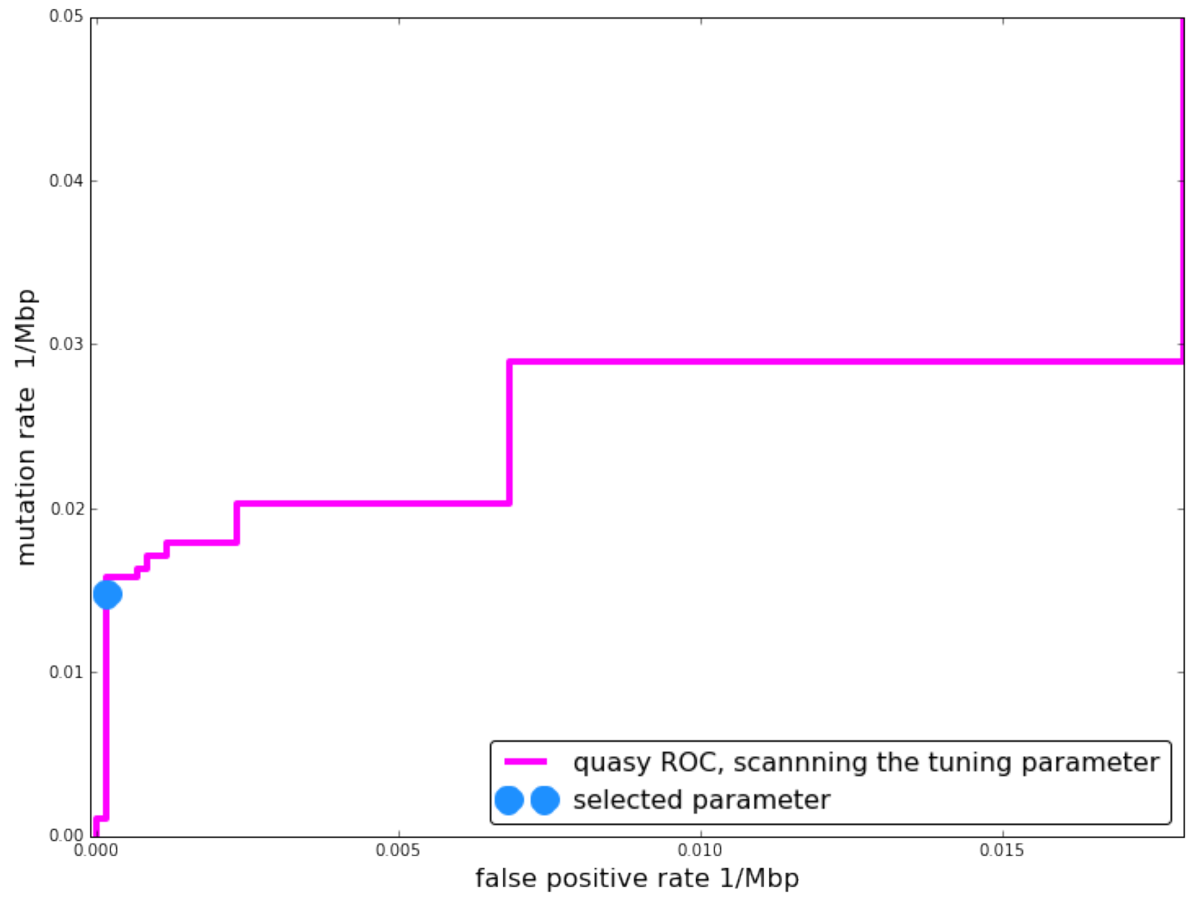

Plotting the final results with optimized score thresholds

In [16]:
```
#define plotting func
def plot_mutres(table):
    #group mutations per samples
    sample_counts=table.groupby(['#sample_idx'],as_index=False).cou
nt()[['#sample_idx','mut']]
    sample_counts.columns=['sample','count']

    #add zeroes if a sample is missing
    N_samples=N
    for i in xrange(N_samples):
        if (not i in set(sample_counts['sample'])):
            sample_counts=pd.concat([sample_counts,pd.DataFrame({'s
ample':[i], 'count' : [0]})])
            sample_counts=sample_counts.sort_values('sample').reset
_index()[['sample','count']]

    #plot
    fig,ax=plt.subplots()
    fig.set_size_inches(12,9)

    #starting clones and controls
    ax.bar(control_idx,[sample_counts.loc[i,'count'] for i in contr
ol_idx],
           facecolor='dodgerblue',edgecolor='none',label='starting
clone and controls')
    #treatment
    ax.bar(not_control_idx,[sample_counts.loc[i,'count'] for i in n
ot_control_idx],
           facecolor='lightgreen',edgecolor='none',label='mutagenic
treatment')

    #samples labels
    names=['DS014','DS026','DS027','DS051','DS052','DS053','DS054',
'DS055','DS056',
          'DS059','DS060','DS061','DS066','DS067','DS068','DS103','D
S104','DS108',
          'DS109','DS110','DS111','DS115','DS116','DS117','DS118','D
S119','DS120',
          'DS121','DS125','DS126','DS141','DS142','DS143','DS144','D
S151','DS152',
          'DS153','DS154','DS155','DS156']

    ax.set_xticks(0.4+np.arange(len(names)))
    ax.set_xticklabels(names,rotation='vertical',fontsize=14)
    #axis, and legend
    ax.set_xlabel(r'samples',fontsize=18)
    ax.set_ylabel(r'Mutations detected',fontsize=18)
    dump=ax.legend(loc='best',fancybox='true',fontsize=16)

    #print the table
    sample_counts['code']=names
    return sample_counts[['code','count']]
```

SNVs

In [17]:
```
plot_mutres(output[(output['type']=='SNV' ) & (output['score']> 1.9
)] )
```

Out[17]:

	code	count
**0**	DS014	1
**1**	DS026	5
**2**	DS027	2539
**3**	DS051	566
**4**	DS052	634
**5**	DS053	485
**6**	DS054	2600
**7**	DS055	2441
**8**	DS056	2201
**9**	DS059	53
**10**	DS060	1241
**11**	DS061	966
**12**	DS066	1
**13**	DS067	43
**14**	DS068	2286
**15**	DS103	967
**16**	DS104	73
**17**	DS108	2
**18**	DS109	491
**19**	DS110	509
**20**	DS111	533
**21**	DS115	2711
**22**	DS116	2904
**23**	DS117	3343
**24**	DS118	2
**25**	DS119	69
**26**	DS120	74
**27**	DS121	94

**28**	DS125	1691
**29**	DS126	1566
**30**	DS141	4
**31**	DS142	70
**32**	DS143	1397
**33**	DS144	351
**34**	DS151	78
**35**	DS152	69
**36**	DS153	2150
**37**	DS154	921
**38**	DS155	362
**39**	DS156	470

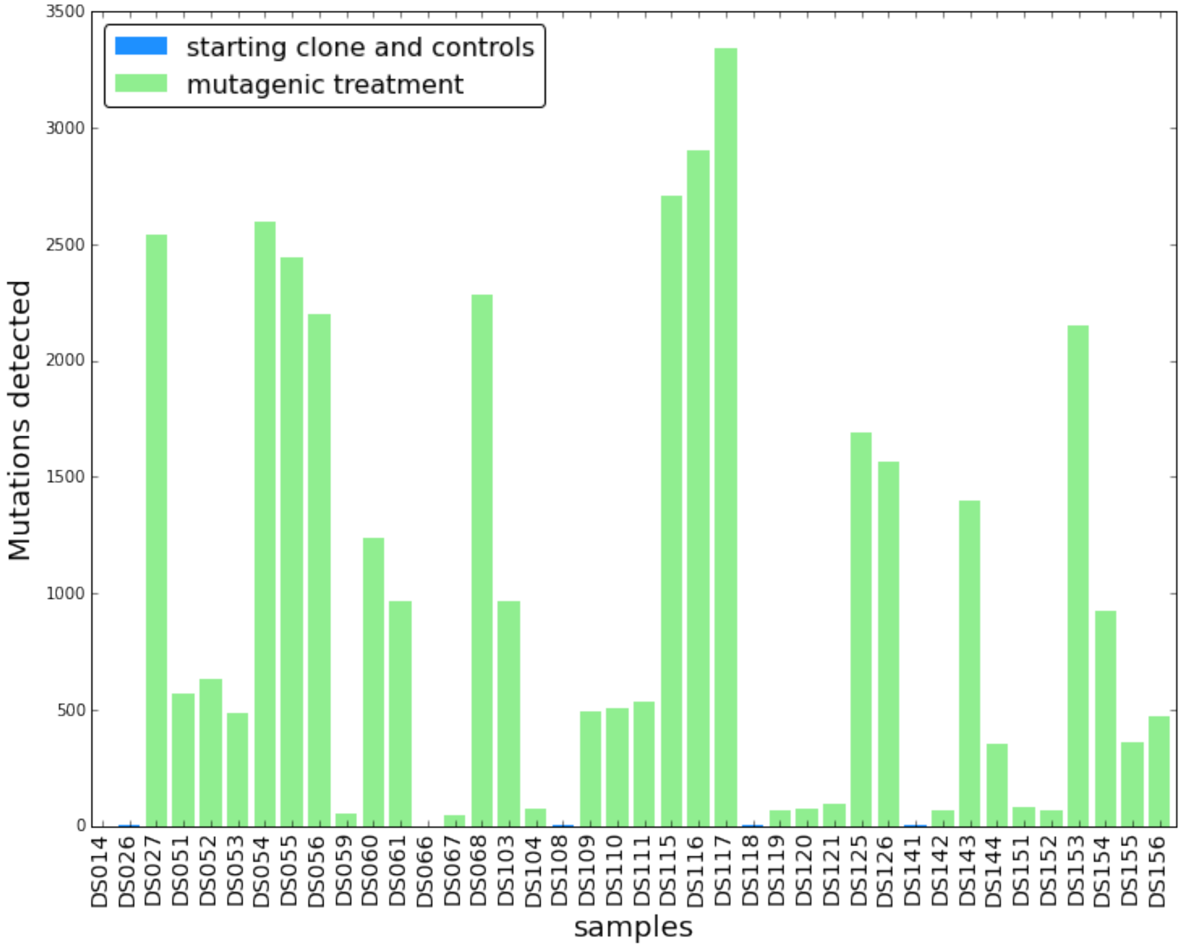

Insertions

In [18]:
```
plot_mutres(output[(output['type']=='INS' ) & (output['score']> 1.7
)] )
```

Out[18]:

	code	count

**0**	DS014	1
**1**	DS026	0
**2**	DS027	1
**3**	DS051	9
**4**	DS052	7
**5**	DS053	8
**6**	DS054	8
**7**	DS055	9
**8**	DS056	5
**9**	DS059	4
**10**	DS060	4
**11**	DS061	4
**12**	DS066	0
**13**	DS067	5
**14**	DS068	3
**15**	DS103	8
**16**	DS104	6
**17**	DS108	0
**18**	DS109	13
**19**	DS110	7
**20**	DS111	11
**21**	DS115	15
**22**	DS116	13
**23**	DS117	7
**24**	DS118	0
**25**	DS119	2
**26**	DS120	2
**27**	DS121	4
**28**	DS125	2
**29**	DS126	5
**30**	DS141	0
**31**	DS142	3
**32**	DS143	2

**33**	DS144	8
**34**	DS151	5
**35**	DS152	6
**36**	DS153	7
**37**	DS154	4
**38**	DS155	11
**39**	DS156	14

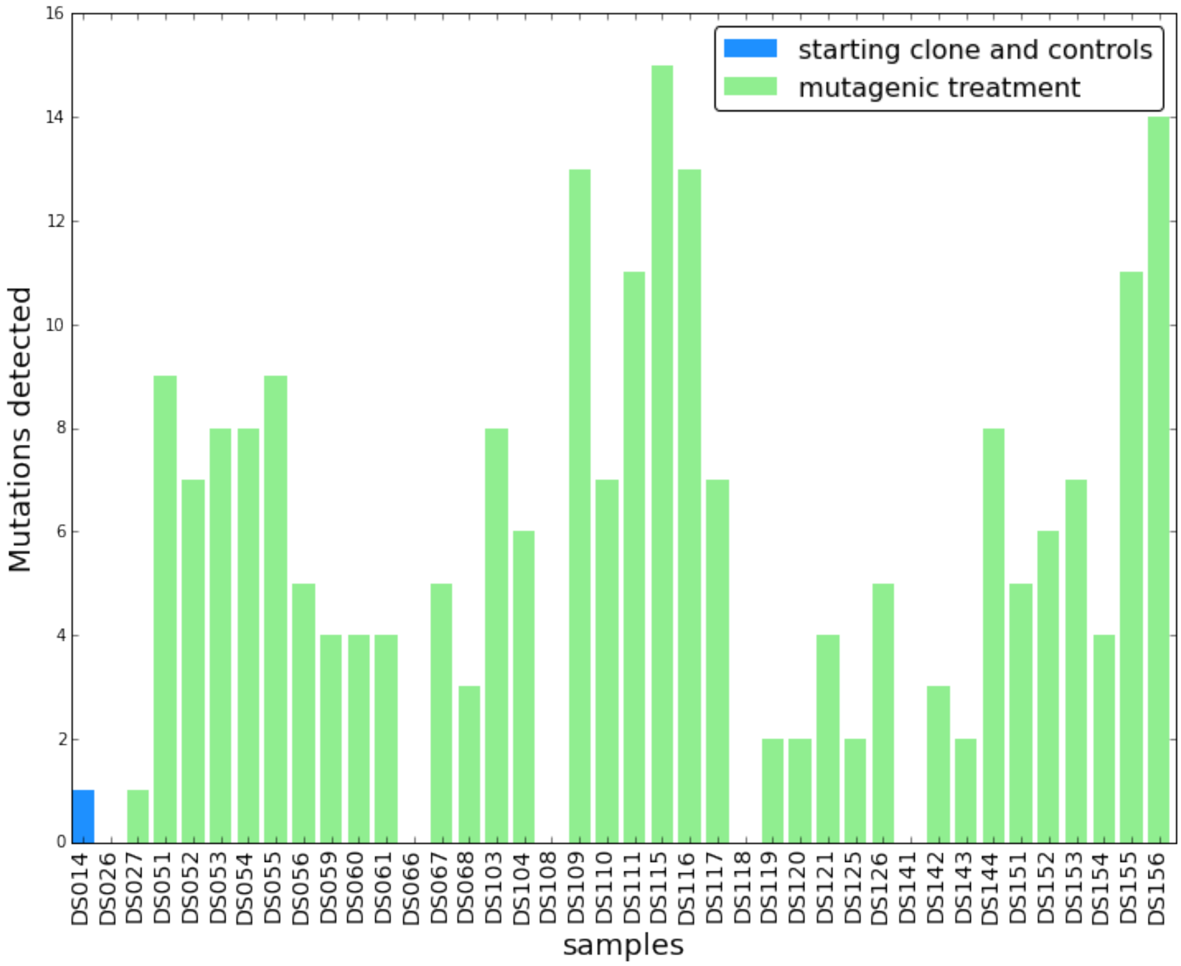

Deletions

In [19]:
```
plot_mutres(output[(output['type']=='DEL' ) & (output['score']>1.8)
] )
```

Out[19]:

	code	count
**0**	DS014	0
**1**	DS026	1
**2**	DS027	9
**3**	DS051	14

**4**	DS052	12
**5**	DS053	12
**6**	DS054	25
**7**	DS055	18
**8**	DS056	31
**9**	DS059	4
**10**	DS060	13
**11**	DS061	6
**12**	DS066	0
**13**	DS067	7
**14**	DS068	6
**15**	DS103	24
**16**	DS104	1
**17**	DS108	0
**18**	DS109	28
**19**	DS110	38
**20**	DS111	33
**21**	DS115	41
**22**	DS116	38
**23**	DS117	42
**24**	DS118	0
**25**	DS119	0
**26**	DS120	4
**27**	DS121	2
**28**	DS125	6
**29**	DS126	12
**30**	DS141	0
**31**	DS142	2
**32**	DS143	5
**33**	DS144	12
**34**	DS151	2
**35**	DS152	1
**36**	DS153	8

**37**	DS154	4
**38**	DS155	20
**39**	DS156	25

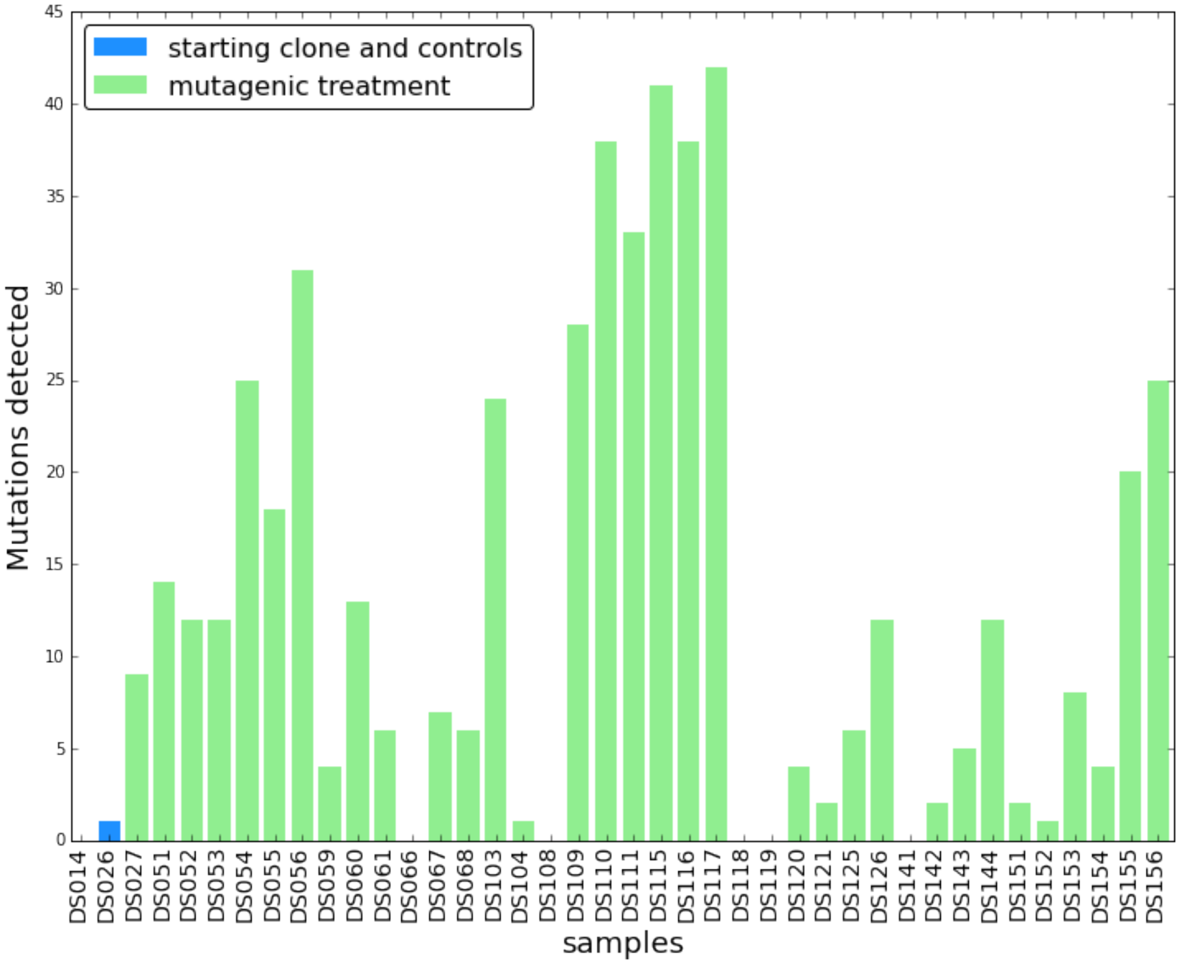

Write the selected results

- download it from here : results (brca_filtered_res.isomut)

```
In [20]: output[(output['type']=='SNV' ) & (output['score']> 1.9) |
                 (output['type']=='DEL' ) & (output['score']>1.8) |
                 (output['type']=='INS' ) & (output['score']> 1.7) ].to_csv(
             '../brca_filtered_res.isomut',index=False, sep='\t')
```

Note: It finished in 16 hours
